# Ca^2+^-dependent regulation of sodium channels Na_V_1.4 and Na_V_1.5 is controlled by the post-IQ motif

**DOI:** 10.1038/s41467-019-09570-7

**Published:** 2019-04-03

**Authors:** Jesse B. Yoder, Manu Ben-Johny, Federica Farinelli, Lakshmi Srinivasan, Sophie R. Shoemaker, Gordon F. Tomaselli, Sandra B. Gabelli, L. Mario Amzel

**Affiliations:** 1 Department of Biophysics and Biophysical Chemistry, Johns Hopkins University School of Medicine, Baltimore, MD 21205 USA; 2 Department of Physiology and Cellular Biophysics, Columbia University, New York, NY 10032 USA; 3 Division of Cardiology, Department of Medicine, Johns Hopkins University School of Medicine, Baltimore, MD 21205 USA; 4 Division of Cardiology, Department of Medicine, Albert Einstein College of Medicine, Bronx, NY 10461 USA; 5 Department of Oncology, Johns Hopkins University School of Medicine, Baltimore, MD 21287 USA

## Abstract

Skeletal muscle voltage-gated Na^+^ channel (Na_V_1.4) activity is subject to calmodulin (CaM) mediated Ca^2+^-dependent inactivation; no such inactivation is observed in the cardiac Na^+^ channel (Na_V_1.5). Taken together, the crystal structures of the Na_V_1.4 C-terminal domain relevant complexes and thermodynamic binding data presented here provide a rationale for this isoform difference. A Ca^2+^-dependent CaM N-lobe binding site previously identified in Na_V_1.5 is not present in Na_V_1.4 allowing the N-lobe to signal other regions of the Na_V_1.4 channel. Consistent with this mechanism, removing this binding site in Na_V_1.5 unveils robust Ca^2+^-dependent inactivation in the previously insensitive isoform. These findings suggest that Ca^2+^-dependent inactivation is effected by CaM’s N-lobe binding outside the Na_V_ C-terminal while CaM’s C-lobe remains bound to the Na_V_ C-terminal. As the N-lobe binding motif of Na_V_1.5 is a mutational hotspot for inherited arrhythmias, the contributions of mutation-induced changes in CDI to arrhythmia generation is an intriguing possibility.

## Abstract (web summary)

Skeletal muscle voltage-gated Na^+^ channel (Na_V_1.4) activity is subject to calmodulin (CaM) mediated Ca^2 +^-dependent inactivation while cardiac Na_V_1.5 is not. Here authors use structural biology, binding and electrophysiology to parse the Ca^2 +^-dependent changes of CaM when bound to the NaV1.4.

## Introduction

Voltage-gated Na^+^ channels (Na_V_s) rapidly activate upon membrane depolarization to allow passage of Na^+^ ions into cells. Of the nine human isoforms of Na_V_, the two isoforms studied here are predominantly expressed in skeletal muscle (Na_V_1.4) and cardiac tissue (Na_V_1.5).

Structurally, Na_V_s are large (~2000 residues) transmembrane proteins composed of four homologous domains (DI-DIV; each containing six transmembrane helices) that form a highly selective Na^+^ pore.^1,2^ DIV is followed by a C-terminal tail which contains a structural domain (CTerm, Na_V_1.4 residues 1599–1754) consisting of a five-helix EF hand-like motif (EFL, helices αI–αV) followed by a long α-helix (helix αVI). Helix αVI contains an IQ (isoleucine-glutamine) motif ([I,L,V]Q**---**R**----**[R,K]), a high-affinity binding domain for calmodulin (CaM) in both its Ca^2+^-free (apo) and Ca^2+^-saturated form^3^

CaM is a small (148 residue) ubiquitous eukaryotic Ca^2+^-sensing regulatory protein. CaM has two homologous lobes (N-lobe, helices A–D, residues 1–76; and C-lobe, helices E–H, residues 81–148) connected by a flexible linker; each lobe is capable of binding 2 Ca^2+^ ions cooperatively. Strong intra-lobe cooperativity means that CaM exists primarily in four states of Ca^2+^-saturation: apo, (Ca^2+^)_2-N_-CaM, (Ca^2+^)_2-C_-CaM and (Ca^2+^)_4_^−^CaM. Each lobe may be closed, semi-open, or open depending on Ca^2+^-saturation. A lobe in the semi-open or open state has a solvent-exposed hydrophobic cleft that may bind target peptides, such as the Na_V_ IQ motif, with high affinity.

Three crystal structures of Na_V_1.5 CTerm/CaM complexes have been reported, each representing a distinct biological state: (1) Na_V_1.5 CTerm in complex with apo CaM (PDB ID: 4OVN^4^), (2) Na_V_1.5 CTerm in complex with apoCaM plus fibroblast growth factor homologous factor (FHF; a Na_V_-inactivating protein, PDB ID: 4DCK^5^), and (3) Na_V_1.5 CTerm in complex with (Ca^2+^)_4_-CaM and FHF (PDB ID: 4JQ0^6^). These structures provide snapshots of the interactions of Na_V_1.5 CTerm and CaM as well as clues to possible CaM binding domains in Na_V_1.4 CTerm; all show the C-lobe bound to the IQ-motif, with the N-lobe in varied configurations. Notably, structure 3 identifies a Ca^2+^-N-lobe binding motif (NLBM) in helix αVI, i.e., a post IQ-motif located past where the C-lobe is bound.

Information regarding the global topology of Na_V_s has been provided by three recent cryo-EM structures of eukaryotic voltage-gated sodium channels: cockroach NavPaS (*P. americana*, PDB ID: 5 × 0 M; 37% identity to *H. sapiens* Na_V_1.4)^7^, eel EeNa_V_1.4 (*E. electricus*, PDB ID: 5XSY; 60% identity to *H. sapiens* Na_V_1.4)^8^ and *H. Sapiens* Na_V_1.4 (PDB ID: 6AGF)^9^. One cytosolic region, the linker between domain DIII and DVI (DIII–DIV linker), has already been implicated in channel regulation. In NavPaS the DIII–DIV linker passes between the CTerm of the channel and the membrane, while in the other two full Na_V_ structures the DIII–DIV linker is visible in a different orientation and no CTerm structure is observed. Previous studies have suggested that an IFM (Ile-Phe-Met) motif in the DIII–DIV linker is the putative fast inactivation gate. Studies have also suggested that the DIII–DIV linker interacts with CaM^10,11^ or Na_V_ CTerm^12^ and the possibility of DIII–DIV linker involvement in Na_V_ 1.4 Ca^2+^ regulation merits consideration.

Na_V_1.4 and Na_V_1.5 have distinct responses to increases in the Ca^2+^ concentration. Na_V_1.4 exhibits a unique CaM mediated Ca^2+^-dependent inactivation (CDI) which results in a reduction of bulk Na^+^ current by approximately 30% after the elevation of cellular Ca^2+^ levels to ~10 µM.^13^ In contrast, Na_V_1.5 exhibits no CDI even at elevated Ca^2+^ levels. It is still not known what are the structural and thermodynamic mechanisms responsible for this difference in behavior are, given the high overall homology between the two Na_V_s (65% identity, 1196 of 1836 residues), especially in the regulatory and CaM-binding CTerm (Na_V_1.4 residues 1599–1754; 78% identity to Na_V_1.5 CTerm).

Here, we present two crystal structures of the Na_V_1.4 CTerm, one bound to apo CaM and the other bound to (Ca^2+^)_4_-CaM, as well as binding data between the CTerm of each isoform and CaM in four states of Ca^2+^-saturation. Comparison of the two crystal structures reveals the Ca^2+^-dependent changes experienced by CaM when bound to the Na_V_1.4 CTerm. The binding data were used to model the populations of four CaM states bound to Na_V_1.4 and 1.5 CTerm as a function of [Ca^2+^] and [CaM]. In addition, electrophysiological data show that the CTerm of Na_V_ controls CDI and that deletion of the post-IQ NLBM in Na_V_1.5 results in robust CDI. These structural, thermodynamic and electrophysiological data all support a mechanism of CDI in Na_V_s controlled by the positioning of the N-lobe of CaM. Ca^2+^-N-lobe binding to the post-IQ NLBM (WT Na_V_1.5) prevents CDI from occurring at elevated Ca^2+^ levels. The absence of a post-IQ NLBM sequence (Na_V_1.4, Na_V_1.5 with NLBM deleted) leads to CDI when the CaM N-lobe binds Ca^2+^. Thus, we showed that the structural and thermodynamic determinants of CDI reside in the CTerm and determine the physiological differences between the two Na_V_ isoforms’ response to Ca^2+^. These findings provide a path to identifying the possibility of Ca^2+^-inactivation in other Na_V_ isoforms.

## Results

### Na_V_ C-terminal tail and CaM N-lobe control CDI

**Fig. 1:**
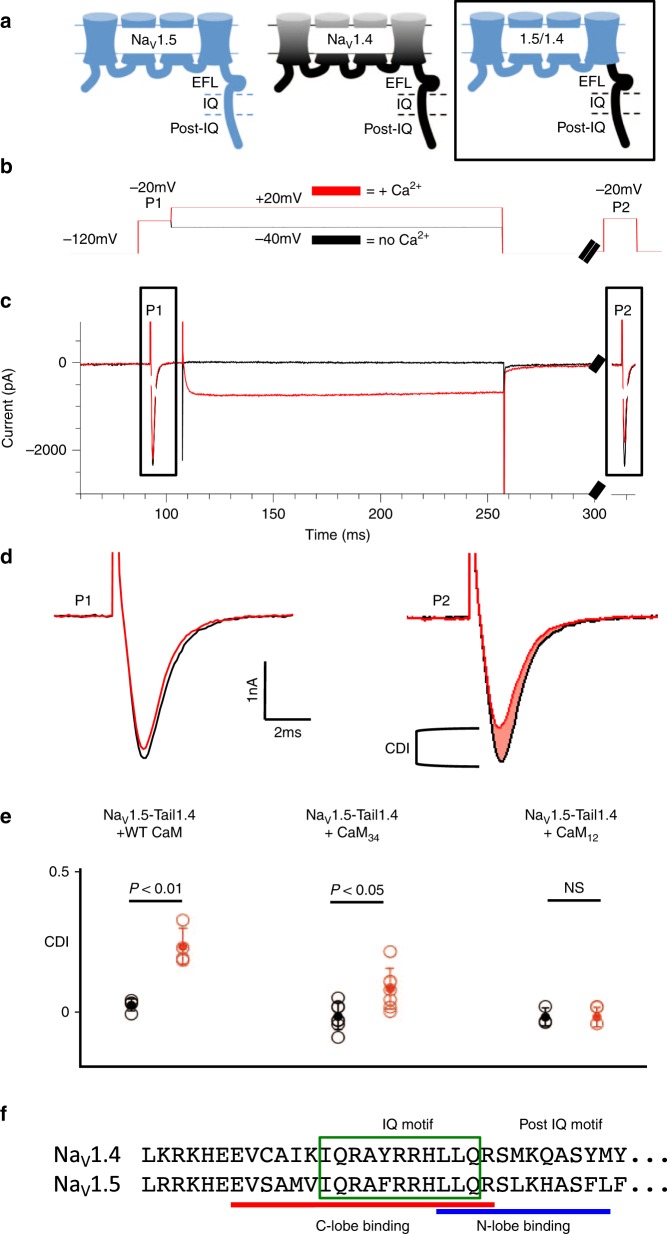
C-terminal tail and CaM control of Na_V_ CDI. **a** Schematic of Na_V_1.5, Na_V_1.4 and Na_V_1.5-CTail1.4 (Na_V_1.5 residues 1–1773 with Na_V_1.4 1599–1836) used in experiments below. **b** Pulse protocol for Na^+^ channel current recordings and assessment of CDI. The pulse protocol with a 150 msec step to +20 mV (red) activates co-expressed Ca_V_2.1 while a step to −40 mV (black) does not. **c** Current elicited by the pulse protocols. The Na^+^ current is probed before (P1) and after (P2) Ca^2+^ entry into the cells due to the intermediate depolarization at −40 mV (where the Ca^2+^ channels are closed) and +20 mV (where the Ca^2+^ channels are open). The pre-pulse P1 and the test pulse P2 are used to probe the Na^+^ current in the absence of Ca^2+^ due to the intermediate depolarization at −40 mV or in the presence of Ca^2+^ due to the activated Ca^2+^ channels during the further depolarization at +20 mV. **d** Na^+^ currents measured during P1 and P2. The P2 current after Ca^2+^ influx (red trace) compared to P2 current with no Ca^2+^ influx (black). **e** Data points of CDI measurement (open circles, filled circle as mean with bars showing ±1 standard deviation) with CaM WT, CaM_12_ or CaM_34_ showing only the N-lobe of CaM is required to bind Ca^2+^ for CDI to occur. Statistical significance was determined by an unpaired t-test. Supplementary Table 2 lists values of the individual data points shown. **f** Alignment of the sequences of Na_V_1.4 and Na_V_1.5 in IQ and post-IQ regions

It was previously shown that Na_V_1.4 responds to an increase in the Ca^2+^ concentration by reducing the maximum Na^+^ current by approximately 30% (calcium-dependent inactivation, CDI)^13^. This effect was observed in HEK293 cells transfected with Na_V_1.4 as well as in skeletal myotubes derived from mouse GLT cells. CDI was not observed in similar experiments involving expressed Na_V_1.5. We performed electrophysiology experiments to confirm the Na_V_-CTerm as the region responsible for the CDI as well as to determine the post-IQ region’s role in physiological Na_V_ function. The sequences of the two isoforms in the IQ and post-IQ regions are shown in Fig. 1f.

Electrophysiology experiments in HEK293 cells were performed using a chimera in which the C-terminal tail of Na_V_1.5 (aa 1774–2016) was replaced by its Na_V_1.4 counterpart (aa 1599–1836). Ca^2+^ was delivered using voltage pulses that selectively activate voltage-gated Ca^2+^-channel Ca_V_2.1 which was co-expressed with the Na_V_ channels. The chimeric protein (Na_V_1.5-CTail1.4; Na_V_1.5 1–1773 with Na_V_1.4 1599–1836) shows CDI, in presence of CaM (Fig. 1a–d). The opposite experiment, Na_V_1.4-CTail1.5, was reported previously and does not show CDI^13^. The participation of CaM as the Ca^2+^ sensor for CDI was confirmed using two engineered CaM mutants with Ca^2+^ binding sites binding disabled in either the N or the C lobe: CaM_12_ (D20A, D56A; N lobe Ca^2+^ binding disabled) or CaM_34_ (D93A, D129A; C lobe Ca^2+^ binding disabled). Only WT CaM and CaM_34_ were capable of inducing CDI (Fig. 1e). These experiments show that CDI is dependent on the Na_V_ C-terminal tail and CaM. Furthermore, the C-lobe of CaM is not required to bind Ca^2+^; the N-lobe binding Ca^2+^ is sufficient to cause CDI.

### Crystal structures of the Na_V_1.4 CTerm–CaM complexes

The structure of the Na_V_1.4 CTerm–CaM complex was determined in the absence and in the presence of 10 mM Ca^2+^. Extensive crystallization trials using two different Na_V_1.4 CTerm constructs—Long (residues 1599–1764) and Short (residues 1599–1754)—produced the crystals used in the structure determinations.

### Crystal structure of Na_V_1.4 CTerm bound to apoCaM

**Fig. 2:**
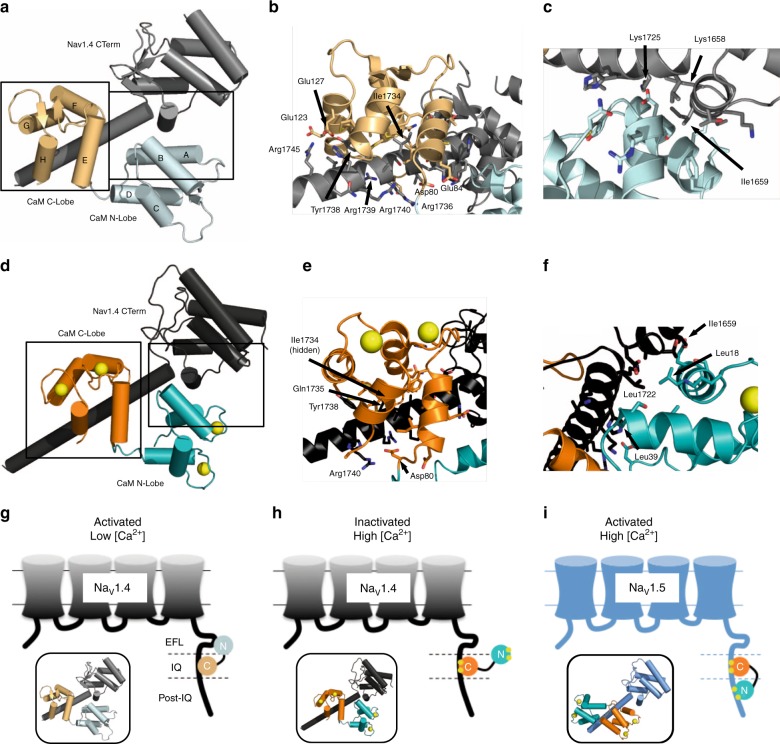
Structures of Na_V_1.4 CTerm in complex with CaM, ± Ca^2+^. **a** 1.8 Å resolution structure of the complex of the Na_V_1.4 CTerm and apo CaM. CTerm colored gray and CaM light teal (N-lobe) and light orange (C-lobe). **b** Close-up of the apo C-lobe and helix α VI interactions. **c** Close-up of N-lobe and EFL interaction. **d** 3.3 Å resolution structure of the complex of the Na_V_1.4 CTerm and (Ca^2+^)_4_-CaM. CTerm is colored black and CaM dark teal (N-lobe) and dark orange (C-lobe). **e** Close-up of Ca^2+^-C-lobe and helix αVI interactions. **f** Close-up of Ca^2+^-N-lobe and EFL interaction. Residues shown have more than 10 Å^2^ BSA. **g–i** Schematics of CaM N-lobe control in Na_V_ regulation. **g** Activated conformation of CaM and Na_V_1.4 (PDB ID: 6MBA). **h** Ca^2+^-inactivated conformation of CaM and Na_V_1.4 (PDB ID: 6MC9). **i** Ca^2+^-insensitive conformation of CaM and Na_V_1.5 (PDB ID: 4JQ0, FHF molecule not displayed)

The 1.8 Å resolution structure of Na_V_1.4 CTerm Long in complex with apo CaM (*R*_work_/*R*_free_ = 20.3/23.4) contains one complex in the asymmetric unit (ASU) of the cell (Fig. 2a, Table 1, Supplementary Fig. 1). The Na_V_1.4 CTerm contains the 5-helix globular EFL (αI–αV, residues Glu1614–Met1706; residues 1599–1613 not observed) followed by a long helix (αVI, residues Leu1722–Lys1748). Helix αVI is in the same orientation relative to the EFL as seen in Na_V_1.5 CTerm in complex with apo CaM (PDB ID: 4OVN)^4^. In contrast, there is a rotation of ~90° of helix αVI relative to the EFL in the structure of Na_V_1.5 CTerm, apo CaM and FHF (PDB ID: 4DCK)^5^. The apo CaM C-lobe is in a semi-open configuration, and bound to the IQ-motif of helix αVI with a buried surface area (BSA) of 872 Å^2^ (Fig. 2b)^14^. The apo C-lobe contacts appear to be anchored by two Na_V_ residues which are in the C-lobe’s hydrophobic pocket: Ile1734 (120 Å^2^ BSA) and Tyr1738 (119 Å^2^ BSA). The CaM C-lobe also forms four Glu–Arg salt-bridges with helix αVI: Glu114–Arg1736, Glu120–Arg1739, Glu123–Arg1745, and Glu127–Arg1745. As expected, in the absence of Ca^2+^ the CaM N-lobe is in a closed conformation. Its surface contacts the CTerm in such a way that CaM helices A and B of the N-lobe contact helices αIII and αVI of the CTerm burying 495 Å^2^ (Fig. 2c). The apo N-lobe contacts are anchored by EFL residue Ile1659 (100 Å^2^ BSA). The CaM N-lobe residue Glu11 is involved in salt-bridges with Na_V_1.4 CTerm: one with Lys1658 and another with Lys1725.

**Table 1:** Data collection and refinement statistics (molecular replacement)

	Na_V_1.4 CTerm + apoCaM PDB ID: 6MBA	Na_V_1.4 CTerm + (Ca^2+^)_4_–CaM PDB ID: 6MC9	Na_V_1.4 CTerm + (Ca^2+^)_4_–CaM Ca^2+^ Anomalous Data
*Data collection*			
Space group			
Cell dimensions	C 1 2 1	P 4_3_ 2_1_ 2	–
* a*, *b*, *c* (Å)	112.7, 29.0, 95.0	72.6, 72.6, 134.7	
α, β, γ (°)	90.0, 123.6, 90.0	90.0, 90.0, 90.0	–
Resolution (Å)	44.87–1.79	40.83–3.30	40.83–3.89
	(1.86–1.79)	(3.42–3.30)	(4.04–3.89)
*R*_merge_	0.072 (0.456)	0.122 (1.64)	0.193 (0.992)
*R*_*pim*_	0.050 (0.312)	0.025 (0.335)	0.040 (0.200)
*I*/σ*I*	8.46 (1.72)	18.11(1.93)	9.95 (2.18)
Completeness (%)	98.0 (96.4)	99.9 (100.0)	99.6 (98.6)
Redundancy	2.9 (3.0)	24.8 (24.5)	23.8 (23.6)
Wavelength (Å)	0.918	0.979	2.515
*Refinement*			
Resolution (Å)	44.87–1.79	40.83–3.30	–
	(1.86–1.79)	(3.42–3.30)	
No. reflections	23,880	5840	–
*R*_work_/*R*_free_	0.203/0.234	0.240/0.285	–
No. atoms			
Protein	2,218	2,326	–
Ligand/ion	20	4	–
Water	120	0	–
*B*-factors (Å^2^)			
Protein	56	146	–
Ligand/ion	55	92	–
Water	47	NA	–
R.m.s. deviations			
Bond lengths (Å)	0.015	0.009	–
Bond angles (°)	1.53	1.06	–

Values in parentheses are for highest-resolution shell

### Crystal structure of Na_V_1.4 CTerm bound to (Ca^2+^)_4_–CaM

The crystal structure of Na_V_1.4 CTerm Short (residues 1599–1754) in complex with (Ca^2+^)_4_–CaM was determined to a resolution of 3.30 Å (*R*_work_/*R*_free_ = 24.0/28.5). Crystals belong to the space group P4_3_2_1_2 and contain one complex in the ASU (Fig. 2d, Table 1). The Na_V_1.4 CTerm shows the same fold observed in the higher resolution structure with apo–CaM with helix α VI in the same orientation which indicates that this helical rotation is insensitive to CaM’s Ca^2+^ state. The (Ca^2+^)-saturated CaM C-lobe bound to the IQ-motif adopts a semi-open^15^ configuration and buries 745 Å^2^ (Fig. 2e), anchored by Ile1734 (103 Å BSA,) and Tyr1738 (123 Å^2^ BSA) as well as Gln1735 (110 Å^2^ BSA). Unlike in the complex with the apo–CaM, in this complex the C-lobe does not form any salt bridges with the Na_V_1.4 CTerm although two residues in the CaM lobe linker are involved in salt bridges: Asp78 and Asp80, both with Arg1740. The N-lobe and EFL interface involves the external regions of the N-lobe, not the hydrophobic cleft, and buries 334 Å^2^ of total area. This interface involves CaM residues Leu18 (84 Å^2^ BSA) and Leu39 (95 Å^2^ BSA) and Na_V_ residues Ile1659 (83 Å^2^ BSA) and Leu1722 (72 Å^2^ BSA).

**Fig. 3:**
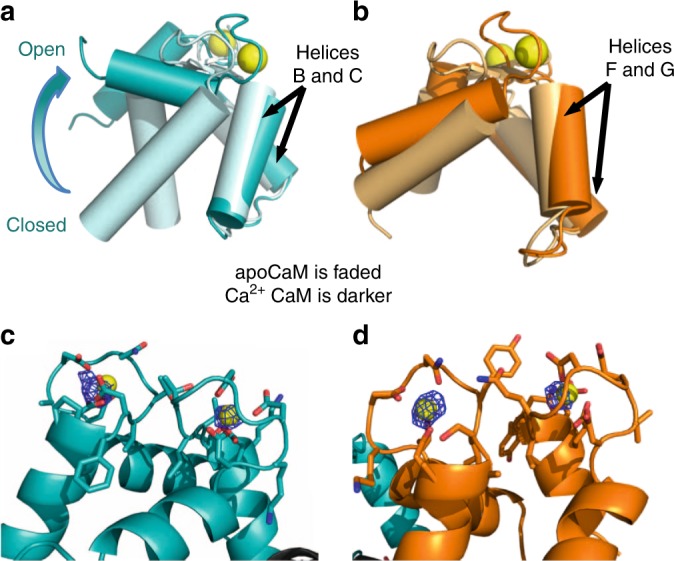
Structural overlap of the N-lobe and C-lobe of CaM bound to Na_V_1.4 CTerm. **a** CaM N-lobes aligned by helices B and C (residues 32–55) show the N-lobe displacement from closed to open upon Ca^2+^ binding (70° of opening). **b** CaM C-lobes aligned by helices F and G (residues 105–128) show the small conformational change (8° of opening) experienced by the C-lobe upon Ca^2+^ binding; both C-lobes are semi-open (Supplementary Fig. 2). **c** Close-up of the C-lobe of (Ca^2+^)_4_-CaM bound to helix α VI, showing the C-lobe’s EF-hand loop residues (CaM residues 93–104 and 129–140) and the anomalous scattering (F_+_ − F_−_)e^iφ-calc^ map at a 4σ contour within 10 Å of either Ca^2+^ ion. **d**, Close-up of the N-lobe of (Ca^2+^)_4_-CaM showing the N-lobe’s EF-hand loop residues (CaM residues 20–31 and 56–67) and the anomalous scattering (F_+_ − F_−_)^eiφ-calc^ map at 3.5σ contour within 10 Å of either Ca^2+^ ion

The Ca^2+^-saturated CaM N-lobe conformation differs significantly from the one seen in the apo–CaM complex (Fig. 2f). The N-lobe’s hydrophobic cleft, formed between helices A–E^16^, is now open and solvent exposed but despite having a 12-residue DIII-DIV linker peptide in the crystallization media, no peptide is observed bound inside the cleft. In contrast, no large conformational change is observed in the CaM C-lobe bound to the Na_V_1.4 CTerm upon Ca^2+^ addition (Fig. 3b and Supplementary Fig. 2). Anomalous scattering was used to confirm the presence of bound Ca^2+^ ions. The same crystal used for structure determination of Na_V_1.4 CTerm bound to (Ca^2+^)_4_–CaM was used to collect data at a wavelength of 2.515 Å, near the anomalous edge of Ca^2+^ (3.070 Å) (Table 1). These data were used to calculate an anomalous map at 3.9 Å resolution. This map showed five peaks above 4σ contour of the map corresponding to the positions of the 4 Ca^2+^ ions, one in each EF-hand loops of CaM (Fig. 3c, d), as well as the sulfur of Met 1668 in the CTerm. This strong anomalous scattering signal confirms the presence of Ca^2+^-with high occupancy in CaM in the crystal and also eliminates the possibility of Ca^2+^-binding sites within the Na_V_1.4 EFL itself, in agreement with previous reports of weak or no-binding between Ca^2+^and Na_V_1.5 EFL^6,17^.

Inspection of crystal packing in the Na_V_1.4 CTerm and (Ca^2+^)_4_–CaM crystal shows an intermolecular interaction between the CTerms of symmetry-related molecules: the end of helix αVI interacts with a groove in the EFL of a neighboring CTerm (Supplementary Fig. 3). The EFL’s groove is formed by helices αΙ, IV, and V with 841 Å BSA. No salt bridge or hydrogen bond interactions are formed in the CTerm and helix αVI interaction and the complex formation significance score (CSS) is 0.00, which strongly suggests that this interaction is a result of crystal packing^14^. It is possible that this interaction is mimicking physiological DIII–DIV linker and EFL interactions (see Discussion).

### Binding measurements of Na_V_ CTerm to CaM

The binding affinities of Na_V_1.4 and Na_V_1.5 CTerms for CaM were determined using isothermal titration calorimetry (ITC). Titrations were conducted under both Ca^2+^-free (with 50 µM EGTA) and Ca^2+^-saturating conditions (with 1 mM CaCl_2_). As CaM has two pairs of EF-hands, Ca^2+^-occupancy of CaM sites is more complex than the binary apo or fully Ca^2+^-saturated captured in these two crystal structures. Each CaM EF hand is capable of binding one Ca^2+^ ion so there are 16 possible states of Ca^2+^-saturation. However, because of the cooperativity between Ca^2+^ binding to the two EF hands in each lobe (i.e., between EF hands 1 and 2 in the N-lobe and between EF hands 3 and 4 in the C-lobe), states in which each lobe is either empty or has 2 Ca^2+^ ions will be the most highly populated. These considerations lead to an approximate model with CaM in 4 states of Ca^2+^-saturation: apo, (Ca^2+^)_2-N_–CaM, (Ca^2+^)_2-C_–CaM and (Ca^2+^)_4_–CaM. Accordingly, binding measurements were performed using CaM_12_ or CaM_34_ under high calcium conditions to capture the CTerm binding affinity for (Ca^2+^)_2-C_–CaM and (Ca^2+^)_2-N_–CaM, respectively. In total, these binding experiments provided data with CaM’s four most physiologically relevant states. Both lengths of the CTerm used in the structural studies were used in the experiments: Long (residues 1599–1764) and Short (residues 1599–1754). Long is considered to reflect physiological binding more accurately and was used in the thermodynamic modeling; Short crystallized with (Ca^2+^)_4_–CaM and Short CTerm binding measurements are also reported here (Supplementary Figs. 4–7). Binding affinities are presented in Table 2. Control titrations using apo CaM_12_ and CaM_34_ were also performed, confirming their function as mimics of half-saturated WT CaM (Supplementary Fig. 8, Supplementary Table 1).

**Table 2:** Binding Data of CaM Species for Na_V_1.4 or Na_V_1.5 CTerm

Sample Cell	[Ca^2+^] mM	Titrant	*K*_d_ nM	−ΔG Kcal mol^−1^
Nav1.4 CTerm Long	0	apoCaM	17 ± 3	10.59
Nav1.4 CTerm Long	1	(Ca^2+^)_4_–CaM	275 ± 26	8.94
Nav1.4 CTerm Long	1	(Ca^2+^)_2-C_–CaM CaM_12_	154 ± 9	9.29
Nav1.4 CTerm Long	1	(Ca^2+^)_2-N_–CaM CaM_34_	121 ± 7	9.43
Nav1.5 CTerm Long	0	apoCaM	48 ± 5	9.98
Nav1.5 CTerm Long	1	(Ca^2+^)_4_–CaM	89 ± 7	9.61
Nav1.5 CTerm Long	1	(Ca^2+^)_2-C_–CaM CaM_12_	3600 ± 200	7.43
Nav1.5 CTerm Long	1	(Ca^2+^)_2-N_–CaM CaM_34_	90 ± 4	9.58
Nav1.4 CTerm Short	0	apoCaM	114 ± 11	9.47
Nav1.4 CTerm Short	1	(Ca^2+^)_4_–CaM	305 ± 21	8.88
Nav1.4 CTerm Short	1	(Ca^2+^)_2-C_–CaM CaM_12_	156 ± 5	9.28
Nav1.4 CTerm Short	1	(Ca^2+^)_2-N_–CaM CaM_34_	282 ± 6	8.93
Nav1.5 CTerm Short	0	apoCaM	39 ± 3	10.10
Nav1.5 CTerm Short	1	(Ca^2+^)_4_–CaM	1520 ± 90	7.93
Nav1.5 CTerm Short	1	(Ca^2+^)_2-C_–CaM CaM_12_	5260 ± 240	7.20
Nav1.5 CTerm Short	1	(Ca^2+^)_2-N_–CaM CaM_34_	376 ± 12	8.76

The *K*_d_s of CaM binding to the skeletal muscle Na_V_1.4 CTerm Long are tighter than 1 µM for all conditions tested (Table 2). Apo CaM displays the highest affinity (*K*_d_ = 17 nM). The intermediate Ca^2+^-saturated states show similar affinity [(Ca^2+^)_2-C_–CaM *K*_d_ = 154 nM, (Ca^2+^)_2-N_–CaM *K*_d_ = 121 nM]. (Ca^2+^)_4_–CaM shows the weakest binding (*K*_d_ = 275 nM) indicating a general trend: the more Ca^2+^-saturated the species of CaM the more they are (modestly) penalized for Na_V_1.4 CTerm binding.

The *K*_d_s of CaM binding to the cardiac Na_V_1.5 CTerm Long range from 48 to 90 nM for three of the species: apoCaM, (Ca^2+^)_2-N_–CaM, and (Ca^2+^)_4_–CaM (Table 2). The fourth CaM species, (Ca^2+^)_2-C_–CaM, binds the Na_V_1.5 CTerm with a *K*_d_ of 3.6 µM, or about 40-fold weaker binding than any other CaM species. The dramatically weakened binding of (Ca^2+^)_2-C_–CaM to Na_V_1.5 CTerm contrasts with the trend observed in Na_V_1.4, for which (Ca^2+^)_2-C_–CaM binds with high affinity to the CTerm. For the Na_V_1.5 CTerm the two tightest binding CaM species are the two physiological endpoint species: apo CaM (*K*_d_ = 48 nM) and fully saturated (Ca^2+^)_4_–CaM (*K*_d_ = 70 nM) which is a trend captured in our thermodynamic model.

### Thermodynamic analysis of CTerm and CaM binding data

**Fig. 4:**
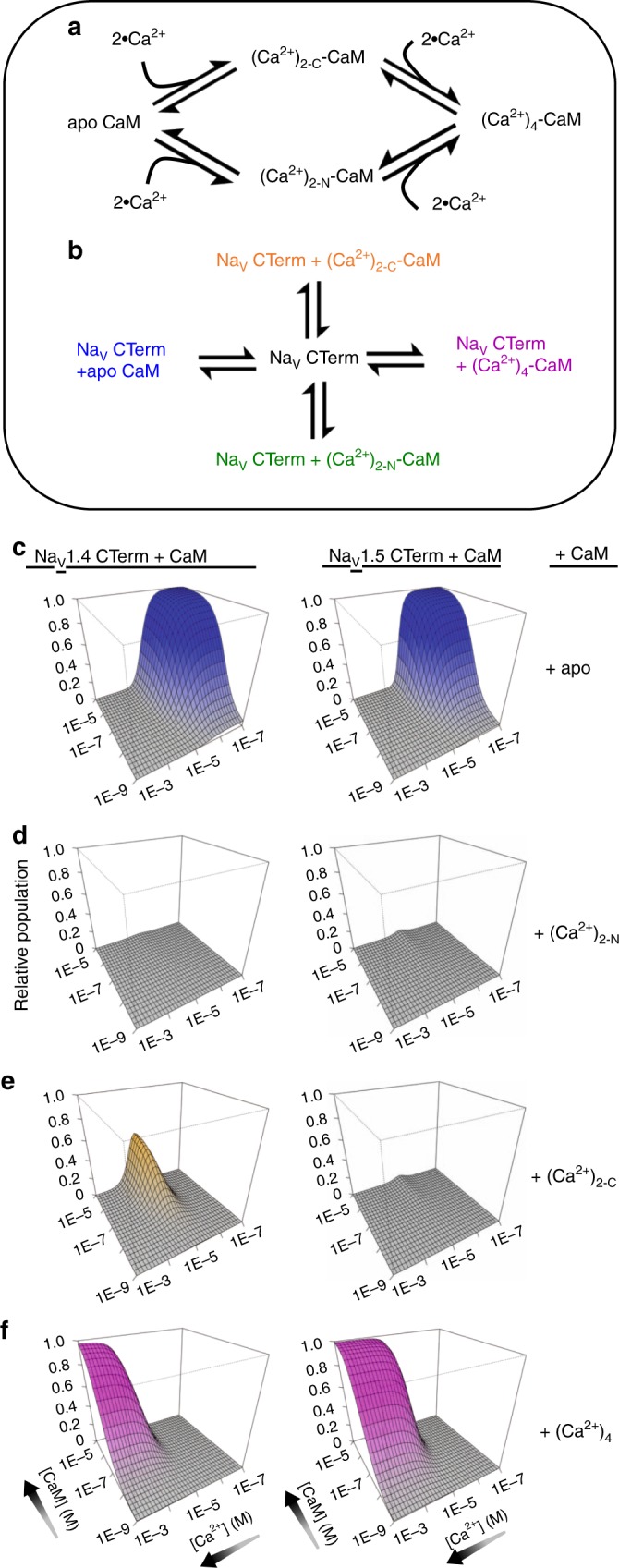
Na_V_1.4 CTerm and Na_V_1.5 CTerm populations with bound CaM. **a** Reaction scheme of Ca^2+^ ions binding to CaM lobes. Thermodynamic data of these binding reactions were reported previously^18, 19^. **b** Reaction scheme of CaM in four Ca^2+^-saturation states binding to Na_V_ CTerm. Thermodynamic data of these binding reactions are reported here. **c–f** Panels showing the relative population (*Z*-axis) of four CaM species bound to Na_V_ CTerm, modeled as a function of [Ca^2+^] and [CaM] using the two schemes above. Na_V_1.4 CTerm-bound species on the left, Na_V_1.5 CTerm-bound CaM-species on the right. **c** Population of apo CaM bound to Na_V_ CTerm. **d** Population of (Ca^2+^)_2-N_–CaM bound to Na_V_ CTerm. **e** Population of (Ca^2+^)_2-C_–CaM bound to Na_V_ CTerm. **f** Population of (Ca^2+^)_4_–CaM bound to Na_V_ CTerm. Only Na_V_1.4 CTerm shows a significant population of bound (Ca^2+^)_2-C_–CaM (orange surface)

The differences in affinity of the two isoforms’ CTerms for the four CaM species must result in different species being present at each Ca^2+^ concentrations and determine the different physiological responses to elevated Ca^2+^ concentration. We employed a thermodynamic model that provides estimates of the populations of Na_V_ CTerm and CaM species at varying [Ca^2+^] and [CaM]. To start, the fractions of the CaM species as a function of [Ca^2+^] can be calculated using the previously reported binding data, which describes Ca^2+^ affinities for the N- and C-lobe of full-length mammalian CaM^18,19^ (Fig. 4a). These affinities provide the necessary information to compute the fraction (*f*_*X*_) of all four species as a function of [Ca^2+^] (Supplementary Fig. 9).

Since the total number of Na_V_ channels is much smaller than the number of CaM molecules, the CaM populations in Fig. 4a will not be affected by binding to the Na_V_ CTerm. In this case, the fractions of five CTerm-CaM species (including free CTerm) can be calculated as a function of the CaM species, using the binding constants reported here (Table 2) and the equations presented in Fig. 4a, b.

**Fig. 5:**
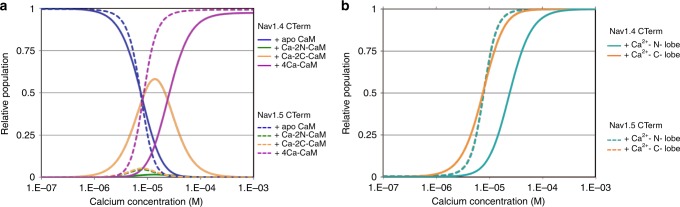
Na_V_ CTerm-CaM populations as a function of Ca^2+^. **a** The relative populations of four CaM-bound CTerm species are shown at a fixed CaM concentration of 10 µM (fixed CaM at 1 µM shows similar trends, Supplementary Fig. 8). The isoform CTerm species add to 100%; free CTerm is included in calculations but not shown in figure. At ~10 µM Ca^2+^ Na_V_1.4 CTerm shows a dominant species of (Ca^2+^)_2-C_–CaM bound to CTerm (solid orange line) while Na_V_1.5 CTerm shows a dominant species of (Ca^2+^)_4_–CaM bound to CTerm (dashed purple line). **b** The relative populations of CTerm-bound CaM, showing Ca^2+^-saturation of the CaM N- or C-lobe. At ~10 M Ca^2+^ CaM bound to Na_V_1.4 CTerm has little (Ca^2+^)_2_-N-lobe (solid teal) while CaM bound to Na_V_1.5 CTerm has primarily (Ca^2+^)_2_-N-lobe (dashed teal line). This is consistent with the hypothesis that Na_V_1.5 CTerm contains an NLBM while Na_V_.14 CTerm does not

This analysis links the concentrations of CaM species, and their [Ca^2+^] dependence, to the species in Fig. 4b. By estimating the fractional population of Na_V_ CTerm species we sidestep working with the concentration of transmembrane Na_V_. This is particularly useful as the 2-D concentration of membrane-bound species is difficult to measure experimentally and the theoretical framework for thermodynamic analysis of 2-D species is less developed. Our model allows the resulting CaM/Ca^2+^/CTerm species populations to be readily calculated from relevant experimental variables and soluble species: [CaM] and [Ca^2+^] (Fig. 4c–f). The Ca^2+^-dependent trends for each isoform are less sensitive to the CaM concentration and are easiest to visualize at constant [CaM] (Fig. 5, Supplementary Fig 10).

### The post-IQ region of Na_V_ controls CDI response

**Fig. 6:**
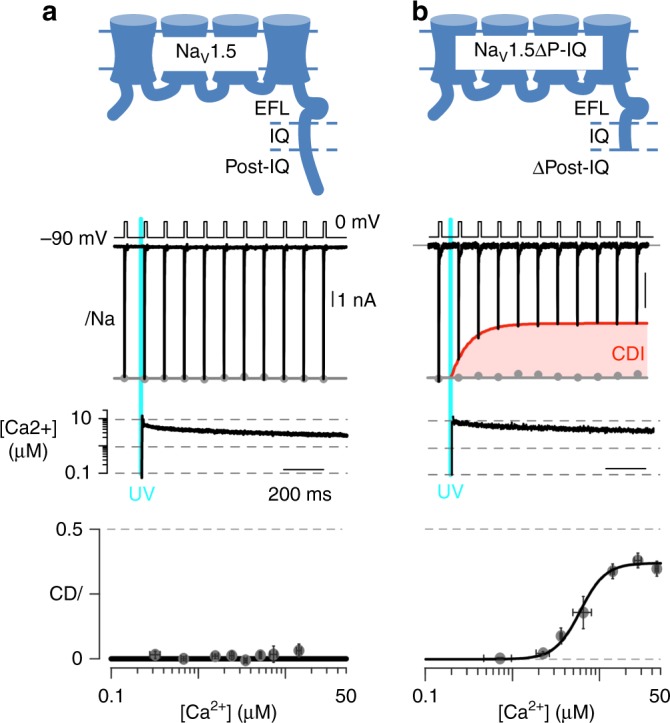
Deletion of Na_V_1.5 post-IQ NLBM reveals CDI. **a** Schematic of the sodium channel Na_V_1.5 displaying CTerm and post-IQ NLBM region; currents unaffected by 10 µM Ca^2+^. Gray dots, peak currents before uncaging. Bottom, mean ± ecm for *CDI* versus Ca^2+^-step amplitude. *CDI* = 1−average peak *I*_Na_ of last three to four responses after Ca^2+^ uncaging/peak *I*_Na_ before uncaging. **b** Schematic of Na_V_1.5 displaying CTerm and the post-IQ deletion; Na^+^ currents reduced strongly at 10 µM Ca^2+^, the envelope of peak currents is outlined in red and the difference in peak current before and after uncaging is shaded. Current measurements of Na_V_1.5ΔP-IQ, shown as in (**a**)

To identify specific regions of the CTerm contributing to the CDI we analyzed the available Na_V_1.5 CTerm and CaM structures. The structure of Na_V_1.5 CTerm in complex with (Ca^2+^)_4_–CaM and FHF (PDB ID: 4JQ0)^6^ identified the post-IQ NLBM as residues 1916–1927 (LLQRSLKHASFL) with the N-lobe binding burying two Na_V_ residues in its hydrophobic cleft: Leu1917 and Leu1921. In contrast, the structures and binding model presented here suggest Na_V_1.4 CTerm lacks such an NLBM. Accordingly, a C-terminally truncated Na_V_1.5 with the post-IQ NLBM removed (Na_V_1.5ΔP-IQ, last residue Ser1920) was used in electrophysiology experiments. This truncation, in the middle of the Ca^2+^-N-lobe binding domain, eliminates the 2^nd^ anchor Leu and any other subsequent downstream Na_V_-CaM contacts. Photo-uncaging Ca^2+^ in HEK293 cells expressing the Na_V_1.5 ΔP-IQ channel leads to robust CDI not observed in full-length Na_V_1.5 (Fig. 6). These experiments are consistent with the Na_V_1.5 post-IQ NLBM preventing the development of CDI in this channel.

## Discussion

Na_V_1.4 exhibits robust regulation by CaM: apo CaM bound to the channel’s CTerm causes an increase in channel open probability^20^ whereas elevated Ca^2+^ (~10 µM) causes the rapid onset of CaM-mediated CDI. In contrast, Na_V_1.5 which also binds CaM in its CTerm, does not exhibit CDI at elevated Ca^2+^^13^. This isoform-specific response has been shown to be CTerm controlled and to require bound CaM. The strongest evidence for the localization of this effect is shown in Fig. 1: a chimera containing the Na_V_1.5 four transmembrane domains followed by the Na_V_1.4 C-terminal tail exhibits robust CDI.

Understanding the different responses of the two isoforms to the increase in the [Ca^2+^] requires the detailed identification of the structural determinants of the CDI as well as the thermodynamics of binding between the two CTerm regions and CaM at different Ca^2+^ concentrations. We carried out binding and structural studies to characterize the interactions of CaM with the Na_V_1.4 CTerm. Apo CaM shows high affinity for Na_V_1.4 CTerm Long (*K*_d_ = 17 nM, Table 2), indicating that CaM is likely bound to the CTerm of Na_V_ at resting Ca^2+^ levels. The structure of apo CaM bound to the Na_V_1.4 CTerm, determined to 1.8 Å resolution, shows that the CaM C-lobe binds to the Na_V_ IQ motif in helix αVI in a semi-open conformation and that the CaM N-lobe makes surface contacts with the CTerm EFL (Fig. 2a–c). This structure is highly similar to that of the complex of Na_V_1.5 CTerm and apo CaM (PDB ID: 4OVN, rmsd 2.71 Å, chains D and I). The similarities between the Na_V_1.4 and Na_V_1.5 CTerm complexes with apo CaM, in both conformation and binding affinities (Na_V_1.5-CTerm Long *K*_d_ = 48 nM, Table 2) and the equivalence of the response of both isoforms to apo CaM binding (i.e., increased activation) suggest that the conformation of the Na_V_1.4-apo CaM crystallized here corresponds to an activating conformation of apo CaM bound to the Na_V_1.4 CTerm.

(Ca^2+^)_4_–CaM binds with high affinity to the Na_V_1.4 CTerm (Table 2), indicating that CaM remains bound to the CTerm at elevated Ca^2+^. The crystal structure of (Ca^2+^)_4_–CaM in complex with Na_V_1.4 CTerm Short shows that the C-lobe of (Ca^2+^)_4_–CaM binds the IQ motif of helix αVI, in a pose similar to that seen in the apo CaM CTerm complex (Fig. 2a, b). In this complex Ca^2+^ binding does not open the C-lobe’s hydrophobic cleft; instead, the C-lobe is fully Ca^2+^-saturated and yet maintains a semi-open configuration (Fig. 3b, d; Supplementary Fig. 2). The structure reported here suggests that the C-lobe serves to anchor CaM to the Na_V_1.4 IQ motif, both in the presence and absence of Ca^2+^, maintaining the same conformation and the same interface.

The N-lobe of Ca^2+^-CaM is found in an open configuration, in contact with the Na_v_ EFL, with a shifted center of mass (3.6 Å shift) and with a reduced BSA relative to the apo CaM N-lobe EFL interface (334 vs. 495 Å^2^). The hydrophobic cleft of the Ca^2+^-saturated N-lobe is open, solvent exposed and unoccupied in this structure (Fig. 3a). Even though the Na_V_1.4 CTerm construct used here includes the entire post-IQ NLBM previously identified in Na_V_1.5 CTerm (Na_V_1.4 residues 1742–1753, LLQRSMKQASYM, 67% identity to Na_V_1.5), no N-lobe binding post-IQ was observed here. This difference indicates that the post-IQ region is crucial for control of Ca^2+^–CaM N-lobe binding.

The largest difference between the affinities of CaM for the Na_V_1.4 and 1.5 CTerm is found in the species with Ca^2+^ only in the C-lobe (Table 2). The (Ca^2+^)_2-C_–CaM_12_ species binds Na_V_1.4 CTerm Long with a *K*_d_ = 154 nM and Na_V_1.5 CTerm Long with a *K*_d_ = 3.6 μM. With CTerm Short the situation is similar: (Ca^2+^)_2-C_–CaM_12_ has a *K*_d_ = 156 nM for Na_V_1.4 and *K*_d_ = 5.26 μM for Na_V_1.5. This large difference may, in part, be responsible for the differences in CDI between the isoforms. Nevertheless, a complete explanation of the differences requires an analysis of all the species contributing to the control of the channel’s activity. To this effect, the binding measurements were used to model the populations of Na_V_ CTerm-CaM species as a function of [CaM] and [Ca^2+^]. Using the experimental affinities, this model provides the relative populations of the five CTerm species in Fig. 2 as a function of [Ca^2+^] and [CaM] that simulate rapid Ca^2+^ signaling in a cell, or the variation of CaM concentration across cells or experiments (Fig. 4)

The populations obtained with this model show similar trends for bound-CaM behavior with both Na_V_ isoforms at the extremes of low or high Ca^2+^ concentration. These trends are most defined at [CaM] of ~10 µM which is near the measured concentration of CaM in cardiac myocytes^21^ and skeletal muscle cells (Fig. 5a).^21,22^ For both isoforms, apo CaM bound to Na_V_ CTerm is the dominant species at physiological resting Ca^2+^ levels (~100 nM) while (Ca^2+^)_4_–CaM bound to Na_V_ CTerm is the dominant species at high Ca^2+^ levels (~100 µM). The isoform populations are most divergent at 10 µM Ca^2+^ at which concentration Na_V_1.4 has a high population of half Ca^2+^-saturated CaM, (Ca^2+^)_2-C_–CaM, while Na_V_1.5 is switching from bound apo CaM to bound (Ca^2+^)_4_–CaM with nearly absent intermediate populations.

This isoform-specific tuning of CaM behavior occurs at a Ca^2+^ concentration that corresponds to those found in relevant cell Ca^2+^ signaling events and which leads to CDI. The half Ca^2+^-saturated species of CaM, (Ca^2+^)_2-C_–CaM, predicted bound to Na_V_1.4 CTerm may then be essential to the molecular mechanism of CDI. However, inspection of the crystal structures presented here shows that Ca^2+^ binding to the C-lobe does not have a major effect on its conformation nor CaM interaction with the Na_V_1.4 CTerm. Instead, it is the lack of Ca^2+^ binding by the N- lobe when the CaM C-lobe is bound to Na_V_1.4 CTerm, at 10 µM Ca^2+^ that is mechanistically relevant. The affinity of Ca^2+^ for the CaM N-lobe is intrinsically low (*K*_d_ ≈ 20 µM) and Ca^2+^ binding is enhanced when bound to a target helix. This lack of Ca^2+^ binding is consistent with a mechanism in which the Na_V_1.4 CTerm lacks a functional NLBM and the N-lobe is free to bind another region of the channel. In contrast, with the Na_V_1.5 CTerm, which does contain the NLBM, the Ca^2+^-N-lobe binds to this motif and is unavailable. This is supported by the thermodynamic binding model which shows that the N-lobe of CaM binds to Na_V_1.5 CTerm more readily when it binds Ca^2+^ (Fig. 5b). If the Ca^2+^-N-lobe does not bind post-IQ it is free to bind some another cytoplasmic region of the Na_V_. This is the interaction that results in CDI. One suggested target for CaM binding in Na_V_ regulation is the cytosolic DIII–DIV linker, (residues 1292–1354 in Na_V_1.4)^23^. In a recent Na^+^ channel structure, NavPaS (PDB ID: 5 × 0 M)^7^ as well as in the Ca_V_1.1 structure (PDB ID: 5GJV)^24^, a portion of the DIII–DIV linker makes contact with the EFL in a pocket formed by helices α I, IV, and V, suggesting the DIII–DIV linker would be near to bound CaM in Na_V_1.4 (Supplementary Fig. 11). Two previous structures, the Na_V_1.5 residues Q1491–1501L bound to the Ca^2+^-saturated C-lobe (PDB ID: 4DJC)^11^ and Na_V_1.5 residues L1514–1522K bound to Ca^2+^-saturated N-lobe (PDB ID: 5DBR)^25^, show helical portions of the Na_V_1.5 DIII–DIV linker (Na_V_1.4 residues 1467–1529) bound to (Ca^2+^)_4_–CaM.

We tested whether the Ca^2+^-N-lobe of CaM attached to the Na_V_1.4 CTerm by the Ca^2+^-C-lobe binding to the IQ motif can bind the DIII–DIV linker. However, attempts at co-crystallizing a peptide that spans residues 1316–1327 of the DIII–DIV linker (first helix) and the Na_V_1.4 CTerm (Ca^2+^)_4_–CaM complex produced crystals but showed no density that could be attributed to the peptide. Furthermore, ITC experiments titrating the Na_V_1.4 CTerm and (Ca^2+^)_4_–CaM complex with two DIII–DIV linker peptides (12 or 20 residues) exhibit no binding even at high peptide concentrations (Supplementary Fig. 12). Binding to the DIII–DIV linker, however, cannot be fully ruled out. Binding as a free peptide *vis a vis* the physiological situation has to overcome two large endergonic processes: (1) stabilization of the helix as required for binding, since the free peptide may not be helical in solution and (2) bringing the peptide and the binder together that in the physiological situation might already be proximal as part of the same complex (chelate effect). Independently of which region is recognized, a proximal binding partner in the Na_V_1.4 for the CaM–Ca^2+^ N-lobe brings together the known crystal structures, the thermodynamic model and the existing physiological data.

The strongest evidence that Na_V_ CDI is controlled via a distal binding partner of the Ca^2+^-N-lobe is provided by electrophysiology experiments using Na_V_1.5. Unlike Na_V_1.4, WT Na_V_1.5 is insensitive to Ca^2+^ concentrations of up to 10 µM and shows no CDI (Fig. 6a). Here we showed that robust CDI may be revealed in Na_V_1.5 by a deletion of the post-IQ NLBM (Fig. 6); that is, elimination of the NLBM results in CDI.

Our results, along with other previously published data, strongly suggest a molecular mechanism of CDI in Na_V_ in which the CTerm and CaM interactions play a crucial role. CaM is anchored by its C-lobe binding to the IQ motif of the Na_V_ CTerm and this interaction is independent of [Ca^2+^]. The N-lobe of CaM acts as a Ca^2+^-dependent switch: it is closed and makes surface contacts with the EFL under apo conditions; upon binding Ca^2+^ the N-lobe switches to the open conformation. The N-lobe is then poised to bind a nearby target in the cytoplasmic regions of the channel. In Na_V_1.5 the Ca^2+^-N-lobe binds the post-IQ NLBM and no CDI results (Supplementary Fig. 13). In Na_V_.14 the N-lobe has a much lower affinity for the post-IQ NLBM and binding to another N-lobe partner results in CDI. Differences in the post-IQ motif between Na_V_1.4 and Na_V_1.5 are likely the cause of this isoform-specific behavior in Ca^2+^-N-lobe binding. Several residues differ between the isoforms in the post-IQ motif (Fig. 1, Supplementary Fig. 14) including the change of Leu1921of Na_V_1.5 to Met (1747) in Na_V_1.4. Leu1921 is buried in the N-lobe’s hydrophobic pocket and makes a close contact with Met73 in Ca^2+^–CaM (PDB ID: 4JQ0). The change of Leu to Met combined with other differences may lead to steric clashes, resulting in a weaker affinity of the Ca^2+^-N-lobe for the post-IQ motif of Na_V_1.4. This proposal identifies a motif that can be used to facilitate the study of Ca^2+^-regulation in other Na_V_ isoforms. Isoform-specific CDI may represent an additional level of regulation of Na_V_ channels reflecting the different characteristics of their currents: periodicity (or not), frequency and duration.

Also, as the post-IQ is a mutational hotspot for inherited arrhythmias, the contributions of mutation-induced changes in CDI to arrhythmia generation is an intriguing possibility^26,27^. The same effects may be operational in neuronal isoforms that mimic the behavior of Na_V_1.5 leading to neurological disorders such as seizures or neuropathic pain.

## Methods

### Human Na_V_1.5-CTail1.4 chimera electrophysiology

The *H. sapiens* Na_V_1.5–eGFP fusion construct was cloned by inserting the coding region of Na_v_1.5 into EGFP-N3 (Clontech)^28^. A chimera was created with Na_V_1.5 up to amino acid 1773 followed by Na_V_1.4 C-terminal tail (residues 1599–1836) and enhanced green fluorescent protein (Na_V_1.5-CTail1.4). Approximately 0.75 × 10^6^ HEK293 cells (American Type Culture Collection, Manassas, VA) were cultured in 6-well tissue culture dishes in Dulbecco’s Modified Eagle Medium (DMEM) supplemented with 10% fetal bovine serum (FBS), L-glutamine (2 mmol/L), penicillin (100 U/mL), streptomycin (10 mg/mL) and gentamicin(50 mg/ml). The cells were co-transfected with plasmids encoding the appropriate Na_V_1.5-CTail1.4, CaM, Ca_V_2.1 EFb 43^+^/44^−^/47^+^ α_2_δ and β_2_α subunits at 1 µg/µl^29,30^. Cells were transfected using LipofectamineTM 2000 (Invitrogen) according to the manufacturer’s instructions and were studied 48 to 72 h post-transfection. The total amount of DNA for all transfections was kept constant.

The bath solution contained (in mM): NaCl, 130; CaCl_2_, 15; MgCl_2_, 1; KCl, 4; NaH_2_PO_4_, 0.33; HEPES, 10; with pH 7.4 adjusted with NaOH and at 290 mOsm adjusted with NaCl. The pipet solution, “0.5 EGTA” contained (in mM): CsMeSO_3_, 124; CsCl, 5; MgCl_2_, 1; MgATP, 4; HEPES (pH 7.4), 10; and EGTA, 0.5; at 290 mOsm adjusted with glucose.

Whole cell *I*_Na_ and *I*_Ca_ were recorded under voltage-clamp with an Axopatch 200 A patch-clamp amplifier (Molecular Devices Corp., Sunnyvale, CA) at room temperature (22 °C). Voltage command protocols were generated by custom-written software and PCLAMP 10 (Molecular Devices Corp). Briefly the protocol is constituted of three parts: a test pulse P_1_ at −20 mV to asses *I*_Na_, steps of 10 mV from −60 mV to +50 mV increase to allow Ca^2+^ to enter the cell and a final test pulse P_2_ at −20 mV to evaluate the effect of Ca^2+^ on *I*_Na_. The length of P1 and P2 were 15 ms, the steps for Ca^2+^ entry were 200 ms in length. The holding potential was −120 mV. Capacitance compensation was optimized and series resistance was compensated by 40–80%. Membrane currents were filtered at 5 kHz and digitized with 12-bit resolution through a DigiData-1200 interface (Molecular Devices Corp.).

### Expression and purification of Na_V_ CTerm–CaM complex

The gene for *H. sapiens* Na_V_1.4 CTerm Long (SCN4A; a.a. 1599–1764; ENF…GDD) in a pGEX-6-P1 plasmid with an N-terminal GST tag was purchased from Genscript, together with the gene for full-length mammalian calmodulin (*R. norvegicus* CALM2; 100% amino acid identity with *H. sapiens* CaM) cloned into a pET24b plasmid^4^ were used to co-transform BL21-CodonPlus RIL *E. coli* (Agilent) cells. The proteins were co-expressed and purified as a complex, using the purification of Na_V_1.5 CTerm and apo CaM as a guide^4^. In brief, the cells were grown overnight at 37 °C in 100 ml of LB medium supplemented with 50 µg/mL kanamycin, 20 µg/mL chloramphenicol and 100 µg/mL ampicillin. Ten mL of the overnight culture was used to inoculate 1 L of LB media containing the same antibiotics. The cells were grown at 37 °C to an absorbance of 0.8 and protein expression was induced with 0.5 mM isopropyl b-D-1-thiogalactopyranoside (IPTG). The cells were grown overnight at 18 °C (approximately 18 h), centrifuged and the cell pellet was frozen at 80 °C.

After thawing, pellets were re-suspended at 0.2 g/mL with phosphate-buffered saline (PBS). DNAse was added and the cells were lysed using a microfluidizer (Microfluidics Corporation; model 110 Y) and the lysate clarified at 27,500 × *g*. The supernatant was loaded a 30 ml Glutathione Sepharose 4 Fast Flow resin using gravity flow. The column was washed with 200 mL buffer and protein was eluted in aliquots of 8 ml with an elution buffer containing 154 mg of reduced L-glutathione in 50 mM Tris-HCl at pH 8. Eluted fractions containing protein were pooled and 5 µg of PreScission protease was added per mg of Na_V_ CTerm and CaM complex. Dialysis was performed against 500 mL of 20 mM Tris, 50 mM NaCl, 1 mM DTT, pH 7.4. The buffer was changed 3 times and the final dialysis was allowed to proceed overnight. The dialyzed and Precission-cleaved protein was loaded on a Source Q anion exchange column. Elution was performed using a buffer of 20 mM Tris and 1 mM DTT and a gradient of 50–500 mM NaCl. Free cleaved GST tag eluted at ~8 mS/cm and Na_V_ CTerm and CaM in complex eluted at ~10 mS/cm. Fractions were judged to be 98% pure by SDS-PAGE gel then pooled and concentrated to ~40 mg/mL protein.

The gene for *Homo sapiens* Na_V_1.4 CTerm Short (SCN4A; aa 1599–1754; ENF…YMY) was cloned into a pGEX-6-P1 plasmid with an N-terminal GST tag. This construct contained an additional 7 residues at the C-terminus, LTRAAAS. BL21-CodonPlus RIL *E. coli* (Agilent) were then co-transformed with Na_V_1.4 CTerm Short and CALM2 pET-24b plasmids^4^. The proteins were co-expressed and purified in complex in the same manner as the Na_V_1.4 CTerm Long and CaM complex.

### Expression and purification of Na_V_ CTerm and CaM

Na_V_1.4 CTerm and CaM were co-expressed in BL21-CodonPlus RIL *E. coli* (Agilent) cells. Cells were grown and lysed as above. The supernatant was loaded onto a Glutathione Sepharose 4 Fast Flow resin using gravity flow column and washed with 1.5 L of low pH PBS buffer with added trifluoroperazine (TFP) and Ca^2+^ (10 mM phosphate, 154 mM NaCl, 1 mM of TCEP, 50 µM TFP, 1 mM CaCl_2_, pH 6.4). This TFP wash removed almost 100% of the CaM bound the Na_V_ CTerm. This was followed by a 4 CV wash of the same buffer lacking TFP and Ca^2+^, a 4 CV wash of PBS at pH 8.0 (10 mM phosphate, 154 mM NaCl, 1 mM of DTT, pH 8.0) and elution with PBS at pH 8.0 containing 10 mM L-glutathione (10 mM phosphate, 154 mM NaCl, 1 mM of DTT, 10 mM L-glutathione, pH 8.0). The Na_V_ CTerm was 70% pure as judged by visual estimation of SDS-PAGE electrophoresis.

The gene for full-length mammalian calmodulin (*H. sapiens/R. norvegicus* CALM2) cloned into a pET24b plasmid^4^ was used for CaM expression. Two engineered CaM mutants, CaM_12_ (D20A and D56A) and CaM_34_ (D93A and D129A), were obtained as a generous gift from the late David Yue lab. CaM_12_ and CaM_34_ are lobe-specific Ca^2+^-binding knockouts have been shown to calcium-insensitive up to 2 mM CaCl_2_^31^. BL21-CodonPlus RIL *E. coli* (Agilent) were transformed with the individual plasmids. In brief, the cells were grown overnight at 37 °C in 100 ml of LB medium supplemented with 50 µg/mL kanamycin and 20 µg/mL chloramphenicol. Ten mL of the overnight culture was used to inoculate 1 L of LB media containing the same antibiotics. The cells were grown at 37 °C to an absorbance of 0.8 and protein expression was induced with 0.5 mM IPTG. The cells were grown overnight at 18 °C (approximately 18 h), centrifuged and the cell pellet was frozen at 80 °C.

After thawing, pellets were re-suspended at 0.2 g/mL with buffer containing 50 mM Tris, 1 mM EDTA, 2 mM DTT, pH 7.4. DNAse was added and the cells were lysed using a microfluidizer (Microfluidics Corporation; model 110 Y) and the lysate clarified at 27,500×*g*. The supernatant was run through at HiPrep™ Phenyl FF (high sub) 16/10 column (GE Lifesciences) equilibrated in a matched buffer using an ÄKTA FPLC (GE Lifesciences). The eluate was collected and brought up to 10 mM CaCl_2_. This high Ca^2+^-eluate was run through a second HiPrep™ Phenyl FF (high sub) 16/10 column (GE Lifesciences) equilibrated in a match high-Ca^2+^ buffer. The column was washed with 200 mL of buffer containing 50 mM Tris, 10 mM CaCl_2_, 2 mM DTT, pH 7.4. Calmodulin was eluted with an elution buffer containing 50 mM Tris, 1 mM EDTA, 2 mM DTT, pH 7.4. Fractions were judged to be 98% pure by SDS-PAGE gel.

### Synthesis of DIII–DIV linker peptides

The 12-mer peptide corresponding to Na_V_1.4 residues 1316–1327 (100% identity with Na_V_1.5 residues 1491–1502) was synthesized with an addition of 2 N-terminal alanines (AAQKKYYNAMKKLG). This peptide has 25% identity with the helical portion of the DIII–DIV linker seen contacting the EFL in NavPaS (PDB ID: 5 × 0 M)^7^. Synthesis was performed by AnaSpec and the peptide was judged to be over 90% pure by HPLC.

The 20-mer peptide corresponding to Na_V_1.4 residues 1312–1331 (100% identity with Na_V_1.5 residues 1487–1506) was synthesized (MTEEQKKYYNAMKKLGSKKP). This peptide has 30% identity with the DIII–DIV linker seen in NavPaS. Synthesis was performed by Genscript and judged to be over 95% pure.

### Structures of Na_V_1.4 CTerm–CaM complexes

The Na_V_1.4 CTerm Long in complex with apo CaM (50 µM EGTA) was crystallized by hanging drop vapor diffusion at 18 °C with a 1:1 ratio of protein to well solution. The well solution contained 0.1 M MES at pH 6.0, 20% PEG 6000 and 1.0 M LiCl. Crystals were flash-frozen in mother liquor prior to data collection. Diffraction data for the crystals were collected on the 17-ID-1 AMX beamline of the NSLS-II (Upton, NY) with a wavelength of 0.918 Å resulting in a 98.0 % complete data set to a resolution of 1.80 Å. Indexing and data reduction were carried out with XDS^32^.

The structure was determined by molecular replacement with Phaser^33^ using as a search model the Na_V_1.5 CTerm–CaM_C_ complex (PDB ID: 4OVN, chain I and D, CaM residues 82–147)^4^ followed by the independent placement of the N-lobe (PDB 1K93, chain D, CaM residues 5–76)^34^. One copy of the CTerm-CaM complex was placed in the asymmetric unit. Model building was completed with iterative cycles of manual rebuilding with Coot^35^ and refinement with Phenix Refine^36^. The structure was refined to an *R*_work_ of 19.9 (*R*_free_ = 23.2) with excellent geometry (Table 1). Buried surface areas (BSA) were calculated with PISA^14^.

The Na_V_1.4 CTerm Short (residues 1599–1754) in complex with CaM was crystallized by hanging drop vapor diffusion at 18 °C with a 1:1 ratio of protein to well solution. Prior to setting the tray, CaCl_2_ and MgCl_2_ were added to a final concentration of 20 mM CaCl_2_, 20 mM MgCl_2_, and the DIII–DIV linker peptide was added at a 1:1 ratio with the CaM-Na_V_1.4 CTerm complex (800 µM). The well solution contained (12.5% w/v PEG 1000, 12.5% w/v PEG 3350, 12.5% v/v MPD, 0.2 M d-glucose, 0.2 M d-mannose, 0.2 M d-galactose, 0.2 M l-fucose, 0.2 M d-xylose, 0.2 M N-acetyl-d-glucosamine 0.1 M MOPS/HEPES-Na pH 7.5; MORPHEUS screen condition F8)^37^. Crystals were flash-frozen in mother liquor prior to data collection. Diffraction data for the crystal were collected at NSLS-II at the 17-ID-2 FMX beamline at a wavelength of 0.979 Å. Diffraction data were then collected from the same crystal at 2.515 Å, near to the Ca^2+^ anomalous edge. Indexing and data reduction were carried out with XDS^32^.

The structure was determined by molecular replacement using the program Phaser^33^ with CaM lobes as search models. Briefly, the coordinates of individual CaM lobes were extracted from PDB depositions containing CaM (at 3.0 Å resolution or better) resulting in 192 N-lobe and 179 C-lobe search models; PDB ID: 2HQW^38^ chain A for the CaM N-Lobe, PDB ID: 3G43^39^ chain B for the CaM C-lobe were the most successful. The CTerm of the Na_V_1.2 CTerm - FHF—(Ca^2+^)_4_–CaM complex (PDB ID: 4JPZ)^6^ was then placed. The crystal contained one copy of the Na_V_1.4 CTerm-CaM complex in the ASU. No density was observed for the DIII–DIV linker peptide. Model building was completed with iterative cycles of manual model building with Coot^35^ and refinement with Phenix Refine^36^. The final structure was refined to an *R*_work_ of 23.8 (*R*_free_ = 29.7) with excellent geometry (Table 1).

### Isothermal calorimetry titration experiments

Titrations were conducted using a VP-ITC MicroCalorimeter (MicroCal Inc.) and data were analyzed with the Origin-5.0 software and fitted to a single binding site per monomer and the error of this fit is reported. The concentration of CTerm used was always between 2 and 25 µM and the concentration of CaM varied from 30 to 1000 µM, depending on the binding affinity. The results of one replicate are reported in Table 2 and Supplementary Table 1. Proteins were prepared for ITC titrations by 3 rounds of dialysis; two of 4-h and one overnight. The buffer used was 20 mM MOPS, 150 mM NaCl, 1 mM MgCl_2_, 50 µM EGTA with 1 mM CaCl_2_ added for high calcium titrations.

A thermodynamic model was built consisting of two sequential reaction schemes. The first reaction (Fig. 4a) is the thermodynamic cycle of CaM binding Ca^2+^. The binding of two Ca^2+^ ions to either CaM lobe was modeled as a single event and single-ion-bound intermediates were not considered. This model contains four states: apoCaM, (Ca^2+^)_2-N_–CaM, (Ca^2+^)_2-C_–CaM, and (Ca^2+^)_4_–CaM. The free energy of cooperative two-site binding of Ca^2+^ to either lobe in full-length mammalian CaM was reported by Evans and Shea as −12.82 ± 0.09 kcal mol^−1^ for the N-lobe and −15.06 ± 0.03 kcal mol^−1^ for the C-lobe and Linse et al. as −12.7 ± 0.6 and −14.9 ± 0.2 kcal mol^−1^^18,19^. Comparing the thermodynamic model results generated with the two sets of binding constants resulted in no discernable difference. The data from Evans and Shea were used in the first binding cycle (Fig. 4a) of the thermodynamic model to match the inclusion of 1 mM MgCl_2_ in our experimental buffer.

### Ca^2+^-uncaging electrophysiology experiments

For whole-cell patch clamp experiments, HEK293 (ATCC CRL-1573) cells were cultured on 10-cm plates, and channels transiently transfected by calcium phosphate method^40^. To construct NaV1.5 ΔP-IQ channel, we PCR amplified the Nav1.5 CT using the forward (AGAGCCAGTGTGAGTCCT) and reverse (agctaatctagattaAGAGCGTTGCAGCAGG) primers (Supplementary Table 3). Subsequently, the amplified DNA segment was digested using KpnI and XbaI restriction enzyme sites and ligated into full-length Nav1.5. For Ca^2+^ uncaging experiments, we applied 8 µg of cDNA encoding either *R. norvegicus* wild type or NaV1.5 ΔP-IQ channel (residues 1–1922), 6 µg of eCFP, and 8 µg of rat CaM_WT_. All of the above cDNA constructs were included within mammalian expression plasmids driven by a cytomegalovirus promoter. To boost expression, cDNA for simian virus 40T antigen (1–2 μg) was co-transfected. Currents were probed ~1–2 days following transfection.

For Ca^2+^-uncaging experiments, internal solution contained (in mM): CsMeSO_3_, 120; CsCl, 5; HEPES (pH 7.4 with CsOH), 10; Fluo-4FF pentapotassium salt (Invitrogen), 0.01; Alexa 568 succinimidyl ester (Invitrogen), 0.0025; Citrate, 1; DM-Nitrophen EDTA (DMN) and CaCl_2_ were adjusted to obtain desired Ca^2+^ flash. Typically, for flashes in range 0.5–2 μM, DMN, 1 mM; and CaCl_2_, 0.7 mM. For the 2–8 µM range, DMN, 2 mM; and CaCl_2_, 1.4 mM. For larger Ca^2+^ steps, DMN, 4 mM; and CaCl_2_, 3 mM. Since DMN can bind Mg^2+^, all experiments were conducted with 0 mM Mg^2+^ internally. The bath solution contained (in mM): TEA-MeSO_3_, 45; HEPES (pH 7.4), 10; NaCl, 100; at 300 mOsm, adjusted with TEA-MeSO_3_.

All Ca^2+^-uncaging experiments were conducted on a Nikon TE2000 inverted microscope with a Plan Fluor Apo 40 × oil objective. Ca^2+^-uncaging was performed using a classic Cairn UV flash photolysis system. UV pulses of ~1.5 ms in duration were powered by a capacitor bank of 4000 µF charged to 200–280 V. PMTs were protected during UV pulse to prevent photo-damage. For simultaneous Ca^2+^ imaging, Fluo4FF and Alexa568 dyes (in fixed, predetermined ratios) were dialyzed into the cell and then probed with Argon laser excitation (514 nm). Autofluorescence for each cell was obtained prior to pipet dialysis of dyes. A field-stop aperture was used to isolate fluorescence from a single cell. Two-color fluorescence measurements were attained using a 545DCLP dichroic mirror, paired with a 545/40BP filter for detecting Fluo4FF, and a 580LP filter for detecting Alexa568. Ca^2+^ measurements were determined from ratio of Fluo4FF/Alexa fluorescence intensities (*R*), according to the relation [Ca^2+^] = *K*_d_ · (*R* − *R*_min_)/(*R*_max_ − *R*). All three parameters *K*_d_, *R*_min_, *R*_max_ were experimentally determined in HEK293 cells dialyzed with reference Ca^2+^ solutions. Briefly, *R*_min_ was determined with internal solution containing 40 mM EGTA, and *R*_max_ using 4 mM Ca^2+^/1 mM EGTA (~3 mM free Ca^2+^) solution. An *R*_20µM_ measurement was obtained with internal solution containing [Ca^2+^] = 20 µM (buffered using NTA). *K*_d_ was experimentally determined by solving the equation above. Calibration measurements were repeated at 1 or 4 mM DMN to account for minor differences in *R*_max_. Uncaging experiments were conducted following 2 min of dialysis of internal solution upon reaching stable current levels. In all cases, the steady-state [Ca^2+^] step amplitude was measured at 150 ms after the instant of uncaging.

### Reporting Summary

Further information on experimental design is available in the Nature Research Reporting Summary linked to this article.

## Supplementary information

Supplementary Information

Reporting Summary

Source Data File

## Data Availability

Data supporting the findings of this manuscript are available from the corresponding authors upon reasonable request. A reporting summary for this Article is available as a Supplementary Information file. The source data underlying Fig. 5a, b, 5e and Supplementary Figs 8 are provided as a Source Data file. Atomic coordinates and structure factors for the Na_V_1.4 CTerm (aa 1599–1754) in complex with (Ca^2+^)_4_–CaM (PDB ID: 6MC9) and for Na_V_1.4 CTerm (aa 1599–1764) in complex with apoCaM (PDB ID: 6MBA) were deposited in the Protein Data Bank.

## References

[1] Payandeh J, Scheuer T, Zheng N, Catterall WA (2011). The crystal structure of a voltage-gated sodium channel. Nature.

[2] Naylor CE (2016). Molecular basis of ion permeability in a voltage-gated sodium channel. EMBO J.

[3] Bahler M, Rhoads A (2002). Calmodulin signaling via the IQ motif. FEBS Lett..

[4] Gabelli SB (2014). Regulation of the NaV1.5 cytoplasmic domain by calmodulin. Nat. Commun..

[5] Wang C, Chung BC, Yan H, Lee SY, Pitt GS (2012). Crystal structure of the ternary complex of a NaV C-terminal domain, a fibroblast growth factor homologous factor, and calmodulin. Structure.

[6] Wang C (2014). Structural analyses of Ca^2+^/CaM interaction with NaV channel C-termini reveal mechanisms of calcium-dependent regulation. Nature communications.

[7] Shen, H. *et al*. Structure of a eukaryotic voltage-gated sodium channel at near-atomic resolution. *Science* **355**, 10.1126/science.aal4326 (2017). 10.1126/science.aal4326 28183995

[8] Yan Z (2017). Structure of the Nav1.4-beta1 complex from electric eel. Cell.

[9] Pan, X. *et al*. Structure of the human voltage-gated sodium channel Nav1.4 in complex with beta1. *Science* **362**, 10.1126/science.aau2486 (2018). 10.1126/science.aau2486 30190309

[10] Sarhan MF, Van Petegem F, Ahern CA (2009). A double tyrosine motif in the cardiac sodium channel domain III-IV linker couples calcium-dependent calmodulin binding to inactivation gating. J Biol Chem.

[11] Sarhan MF, Tung CC, Van Petegem F, Ahern CA (2012). Crystallographic basis for calcium regulation of sodium channels. Proc. Natl Acad. Sci. USA.

[12] Gardill BR (2018). The voltage-gated sodium channel EF-hands form an interaction with the III-IV linker that is disturbed by disease-causing mutations. Sci. Rep..

[13] Ben-Johny M (2014). Conservation of Ca2+/calmodulin regulation across Na and Ca2+channels. Cell.

[14] Krissinel E, Henrick K (2007). Inference of macromolecular assemblies from crystalline state. J. Mol. Biol..

[15] Kawasaki H, Kretsinger RH (2014). Structural differences among subfamilies of EF-hand proteins--a view from the pseudo two-fold symmetry axis. Proteins.

[16] Babu YS, Bugg CE, Cook WJ (1988). Structure of calmodulin refined at 2.2 A resolution. J. Mol. Biol.

[17] Kim J (2004). Calmodulin mediates Ca2+sensitivity of sodium channels. J. Biol. Chem..

[18] Evans TI, Shea MA (2009). Energetics of calmodulin domain interactions with the calmodulin binding domain of CaMKII. Proteins.

[19] Linse S, Helmersson A, Forsen S (1991). Calcium binding to calmodulin and its globular domains. J. Biol. Chem..

[20] Adams PJ, Ben-Johny M, Dick IE, Inoue T, Yue DT (2014). Apocalmodulin itself promotes ion channel opening and Ca^2+^ regulation. Cell.

[21] Klee CB, Vanaman TC (1982). Calmodulin. Adv Protein Chem.

[22] Maier LS (2006). Dynamic changes in free Ca-calmodulin levels in adult cardiac myocytes. J. Mol. Cell. Cardiol..

[23] The UniProt, C. (2017). UniProt: the universal protein knowledgebase. Nucleic. Acids. Res..

[24] Wu J (2016). Structure of the voltage-gated calcium channel Ca(v)1.1 at 3.6 A resolution. Nature.

[25] Johnson, C. N. et al. A mechanism of calmodulin modulation of the human cardiac sodium channel. *Structure*, 10.1016/j.str.2018.03.005 (2018). 10.1016/j.str.2018.03.005 PMC5932218 29606593

[26] Ackerman MJ, Clapham DE (1997). Ion channels--basic science and clinical disease. N. Engl. J .Med..

[27] Loussouarn G (2015). Physiological and Pathophysiological Insights of Nav1.4 and Nav1.5 Comparison. Front. Pharmacol..

[28] Biswas S, DiSilvestre D, Tian Y, Halperin VL, Tomaselli GF (2009). Calcium-mediated dual-mode regulation of cardiac sodium channel gating. Circ. Res..

[29] Ben Johny M, Yang PS, Bazzazi H, Yue DT (2013). Dynamic switching of calmodulin interactions underlies Ca2+regulation of CaV1.3 channels. Nat. Commun..

[30] Chaudhuri D (2004). Alternative splicing as a molecular switch for Ca2+/calmodulin-dependent facilitation of P/Q-type Ca2+channels. J. Neurosci.

[31] Shao D (2014). The individual N- and C-lobes of calmodulin tether to the Cav1.2 channel and rescue the channel activity from run-down in ventricular myocytes of guinea-pig heart. FEBS letters.

[32] Kabsch W (2010). Xds. Acta. Crystallogr. D Biol Crystallogr..

[33] McCoy AJ (2007). Phaser crystallographic software. J. Appl. Crystallogr..

[34] Drum CL (2002). Structural basis for the activation of anthrax adenylyl cyclase exotoxin by calmodulin. Nature.

[35] Emsley P, Lohkamp B, Scott WG, Cowtan K (2010). Features and development of Coot. Acta .Crystallogr. D Biol. Crystallogr..

[36] Adams PD (2010). PHENIX: a comprehensive Python-based system for macromolecular structure solution. Acta. Crystallogr. D Biol. Crystallogr..

[37] Gorrec F (2009). The MORPHEUS protein crystallization screen. J. Appl. Crystallogr..

[38] Ataman ZA, Gakhar L, Sorensen BR, Hell JW, Shea MA (2007). The NMDA receptor NR1 C1 region bound to calmodulin: structural insights into functional differences between homologous domains. Structure.

[39] Fallon JL (2009). Crystal structure of dimeric cardiac L-type calcium channel regulatory domains bridged by Ca2+* calmodulins. Proc. Natl Acad. Sci. USA.

[40] Peterson BZ, DeMaria CD, Adelman JP, Yue DT (1999). Calmodulin is the Ca2+sensor for Ca2+-dependent inactivation of L-type calcium channels. Neuron.

