# Supplementary material for: Ca2+-dependent regulation of sodium channels NaV1.4 and NaV1.5 is controlled by the post-IQ motif
Source: Nat Commun. 2019 Apr 3;10:1514. doi: 10.1038/s41467-019-09570-7 (PMC6447637; doi:10.1038/s41467-019-09570-7)
Supplement: Supplementary file 1 — Supplementary Information [file 41467_2019_9570_MOESM1_ESM.pdf]

Ca^{2+}-Dependent Regulation of Sodium Channels $Na_V1.4$ and $Na_V1.5$ is Controlled by the Post-IQ Motif

Yoder et al

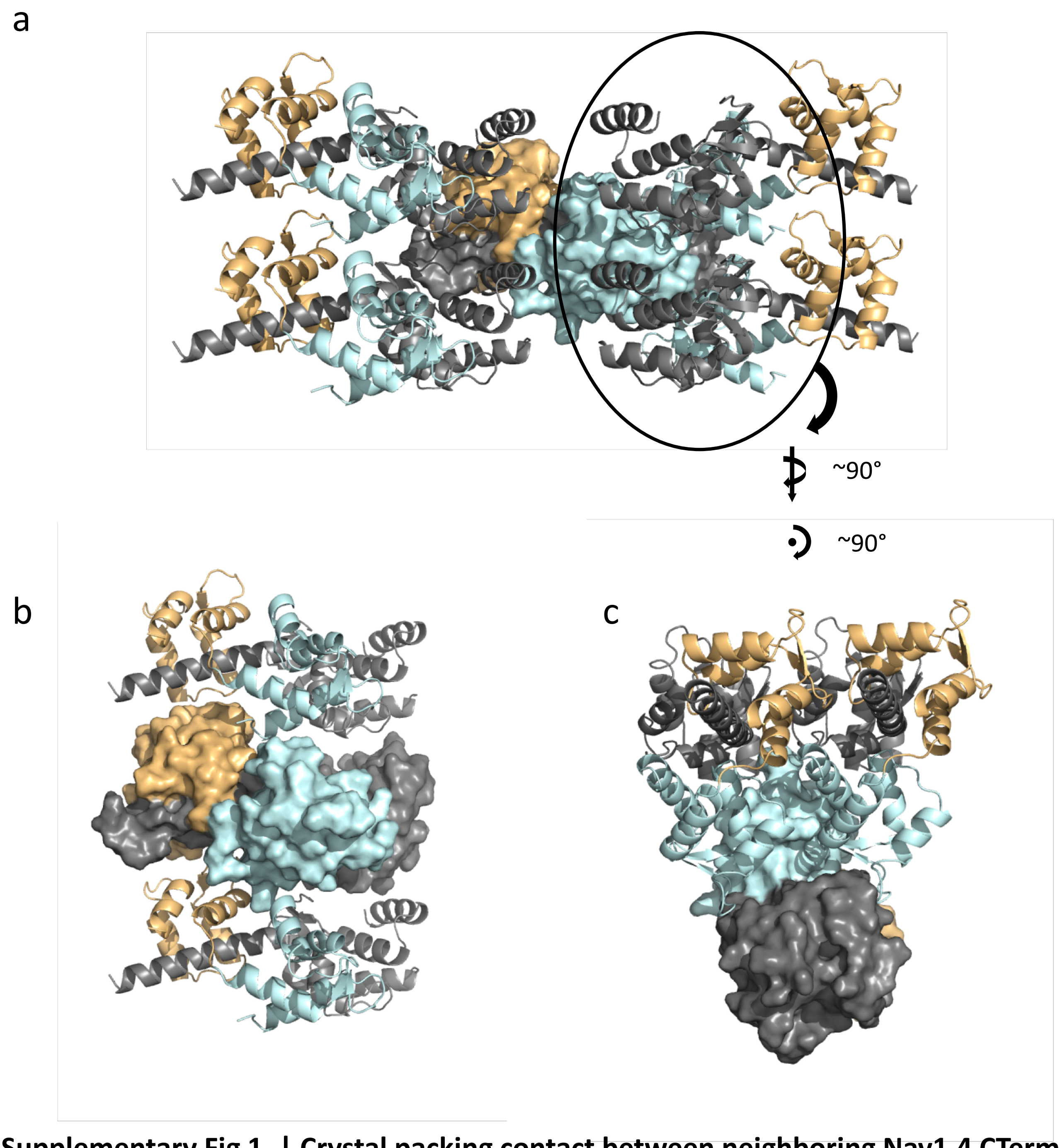

Supplementary Fig 1. | Crystal packing contact between neighboring Nav1.4 CTerm and apo CaM complexes. Reference copy is shown in surface and contacting neighbors in cartoon. **a,** Top view of crystal packing contacts of N-lobe of CaM include 4 CTerms of neighbors. **b,** Side view shows two other neighbors have contact through N-lobe to N-lobe interfaces. **c,** Side view of (**a**) shows that two neighboring complexes that make contact with CTerm also make contact with N-lobe to N-lobe interface.

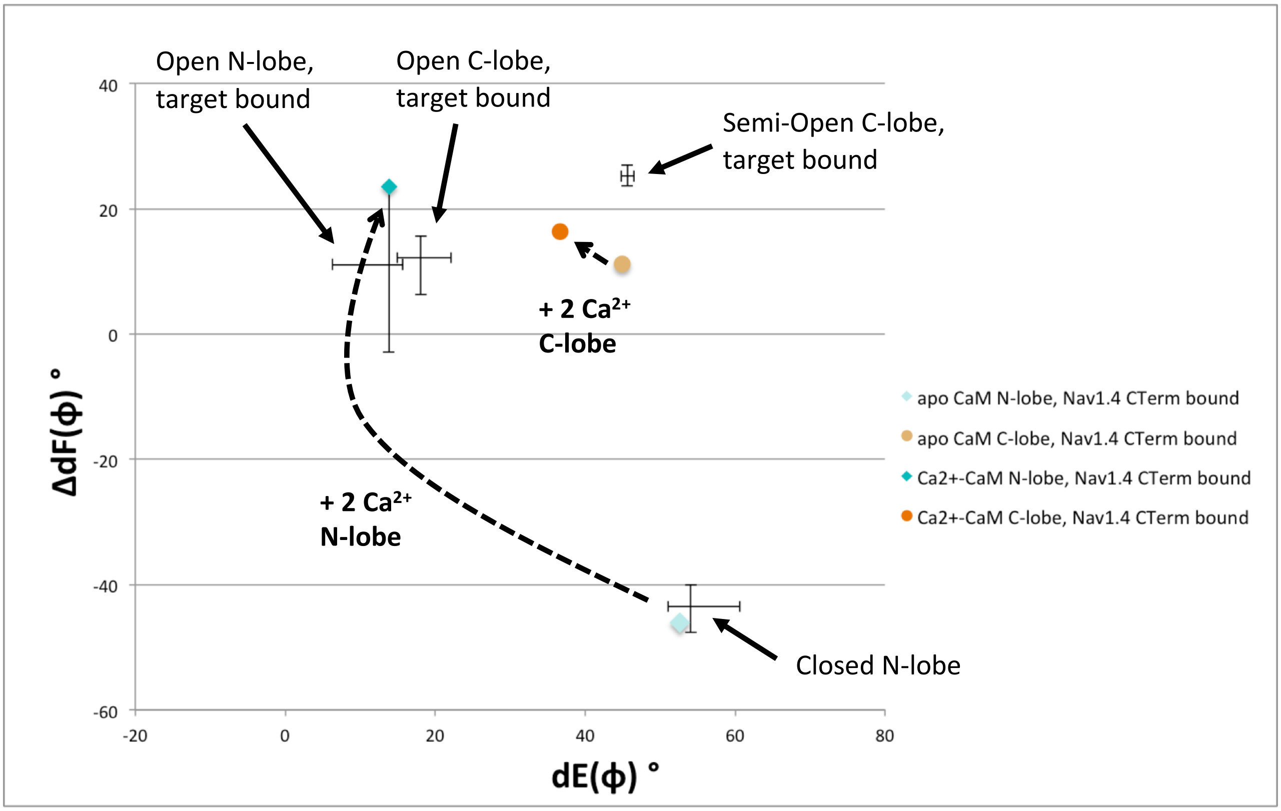

Supplementary Fig. 2 | Classification of calmodulin lobe conformation. (Redrawn from Fig 4 in [1]. The helical angles of a CaM lobe are used to classify the lobe's conformation. dE(Φ) is the azimuthal angle between the y-axis and the projection of the offset vector to helix E onto the yz-plane, and ΔdF(Φ) is the difference angle between the two helices (F1 and F2) of odd and even domains in EF hand lobes. Crosses are centered at median value ± 1st and 3rd quartile range for CaM lobes found in PDB database, as previously reported [1]. In CaM bound to $Na_V1.4$ CTerm, there is little change in C-lobe conformation upon Ca^{2+} binding and the lobe remains best described as semi-open. The N-lobe experiences a large conformational change upon Ca^{2+} binding.

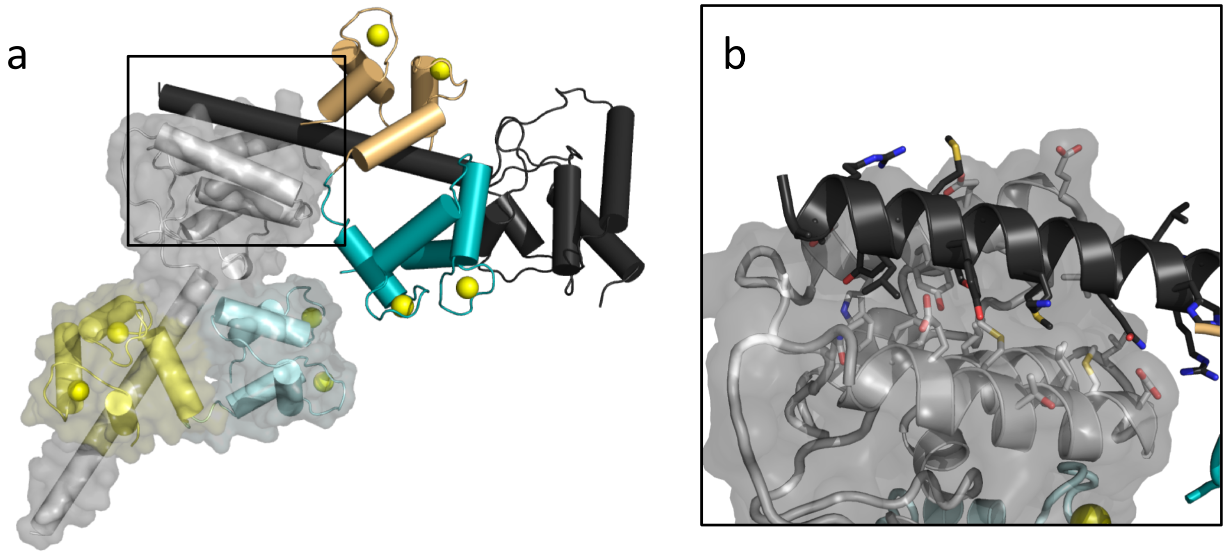

Supplementary Fig 3. | Crystal packing contact between neighboring Nav1.4 CTerms in the Nav1.4 CTerm and $(Ca^{2+})_4$-CaM complex. a, Two crystallographic symmetry mates of Na_V1.4 CTerm and $(Ca^{2+})_4$-CaM showing helix αVI (black) and EFL (gray) contact between neighbors. This interaction may mimic DIII-DIV linker -EFL contact observed in NavPaS [2]. **b,** Close-up of helix α VI -EFL contact.

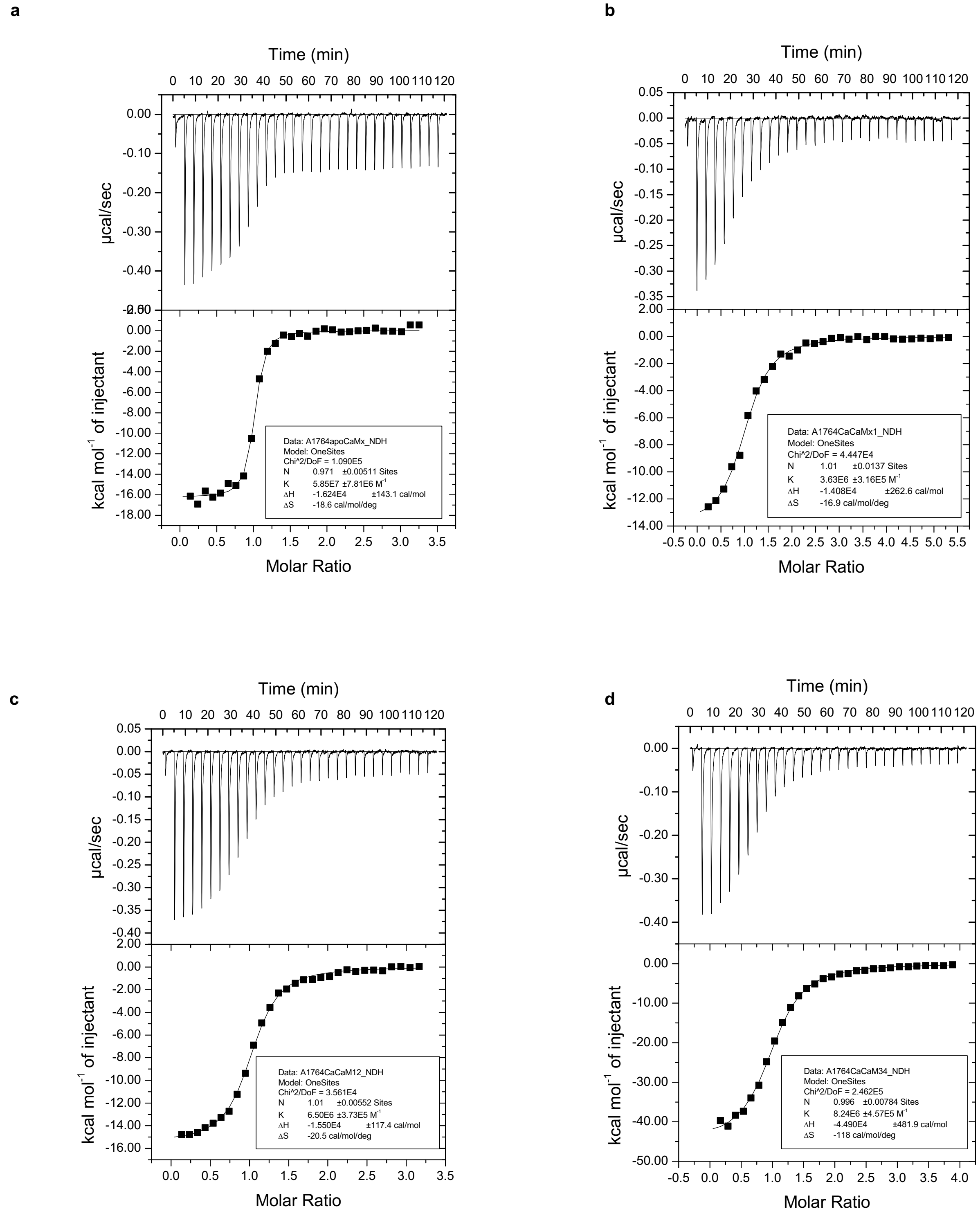

Supplementary Fig. 4 | $Na_V1.4$ CTerm long and CaM binding.
Isotherms of ITC titrations, top panel displays the heat evolved following each injection and the bottom panel shows the integrated heats of injection. **a,** apo CaM (43 µM) into $Na_V1.4$ CTerm long (3.5 µM). **b,** $(Ca^{2+})_4$-CaM (77 µM) into $Na_V1.4$ CTerm long (3.2 µM). **c,** $(Ca^{2+})_{2\text{-}C}$-CaM (66 µM) into $Na_V1.4$ CTerm long (4.8 µM). **d,** $(Ca^{2+})_{2\text{-}N}$-CaM (29 µM) into $Na_V1.4$ CTerm long (2.9 µM).

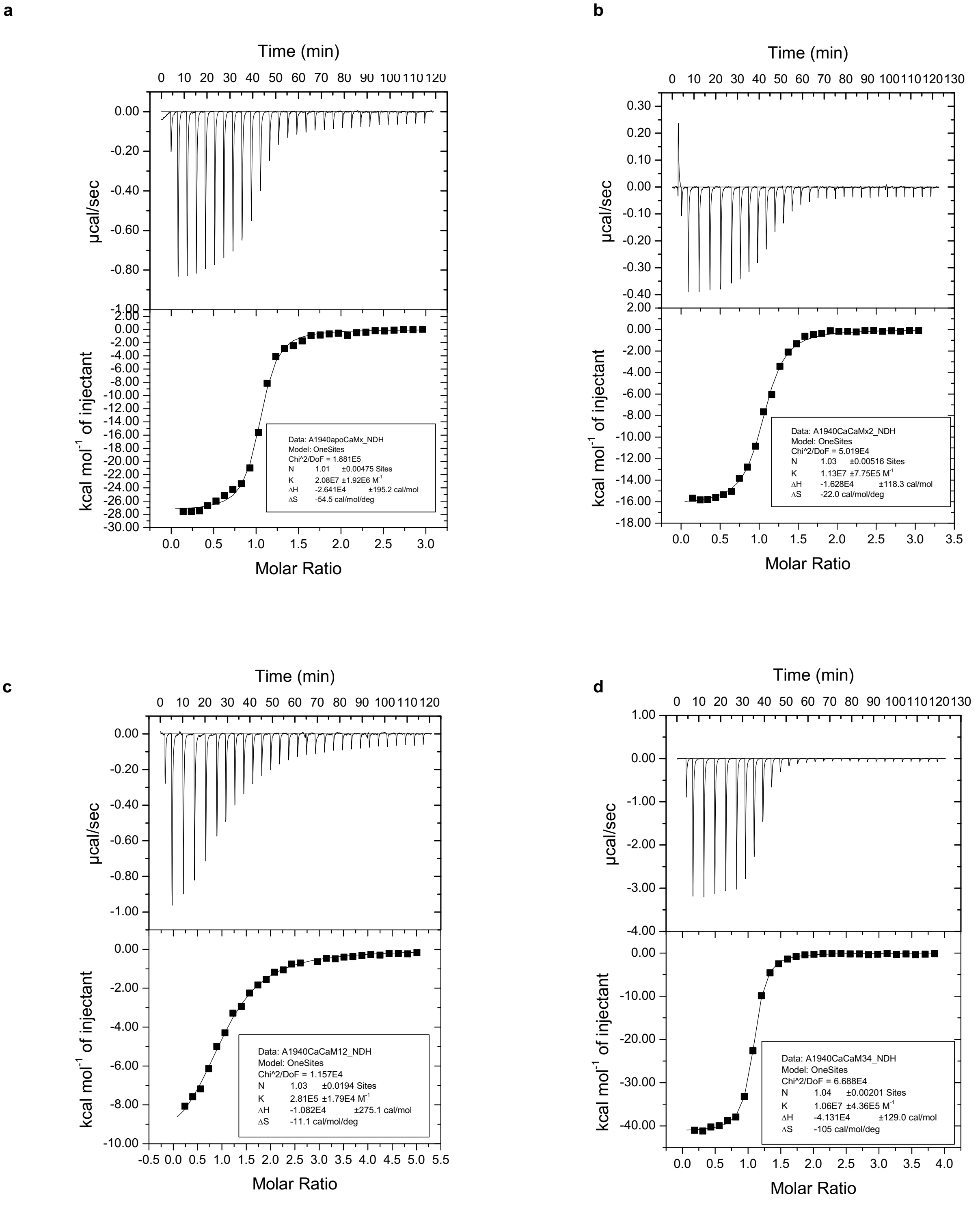

Supplementary Fig. 5 | Na_V1.5 CTerm Long and CaM Binding.
Isotherms of ITC titrations, top panel displays the heat evolved following each injection and the bottom panel shows the integrated heats of injection. **a,** apo CaM (74 µM) into Na_V1.5 CTerm long (5.5 µM). **b,** $(Ca^{2+})_4$-CaM (65 µM) into Na_V1.5 CTerm long (4.7 µM). **c,** $(Ca^{2+})_{2\text{-}C}$-CaM (330µM) into Na_V1.5 CTerm long (14.5 µM). **d,** $(Ca^{2+})_{2\text{-}N}$-CaM (201 µM) into Na_V1.5 CTerm long (12 µM).

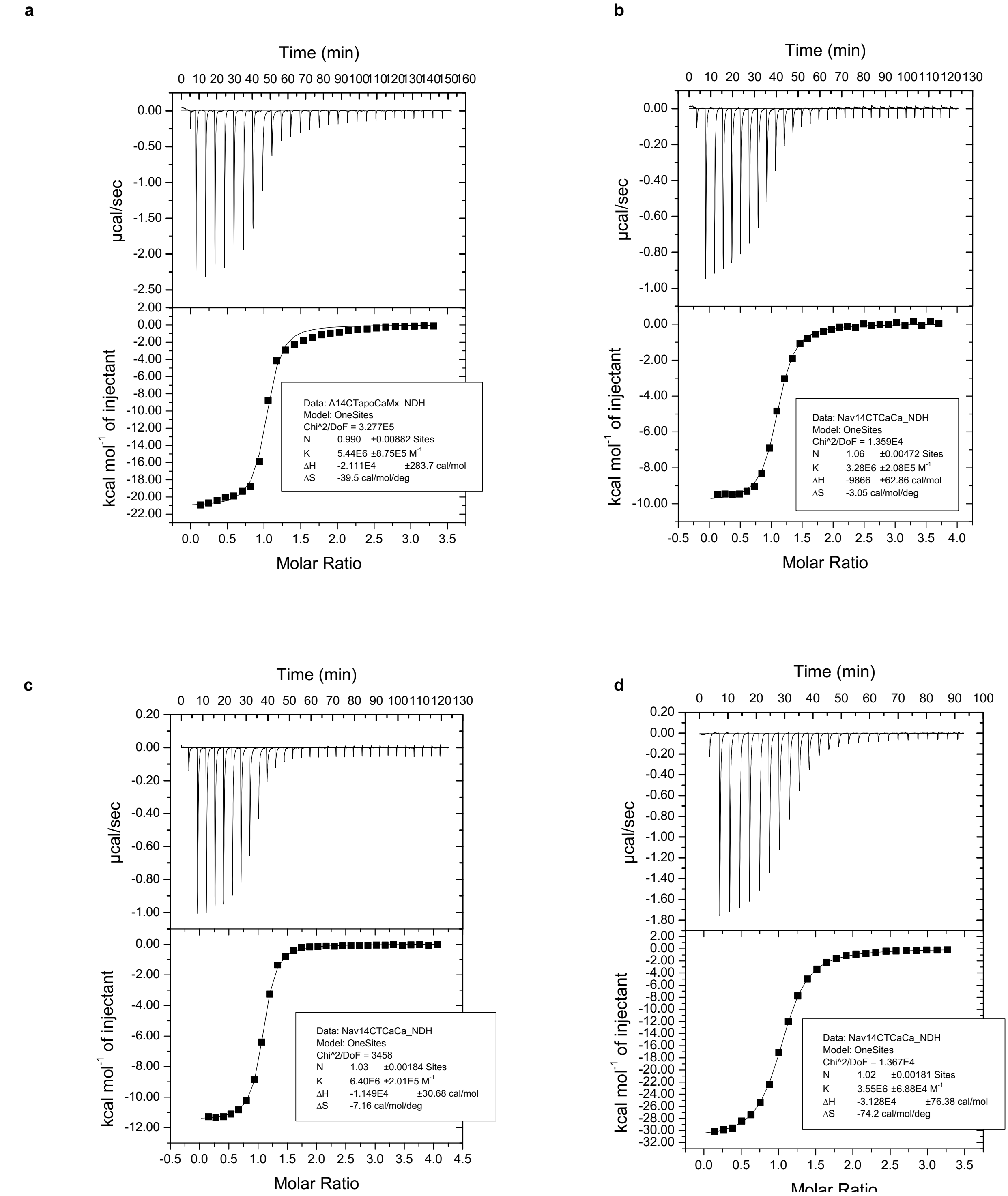

Supplementary Fig. 6 | Na_V1.4 CTerm Short and CaM Binding. Isotherms of ITC titrations, top panel displays the heat evolved following each injection and the bottom panel shows the integrated heats of injection. **a,** apo CaM (285 µM) into Na_V1.4 CTerm short (18 µM). **b,** $(Ca^{2+})_4$-CaM (279 µM) into Na_V1.4 CTerm short (16 µM). **c,** $(Ca^{2+})_{2\text{-}C}$-CaM (252 µM) into Na_V1.4 CTerm short (14 µM). **d,** $(Ca^{2+})_{2\text{-}N}$-CaM (158 µM) into Na_V1.4 CTerm short (9.3 µM).

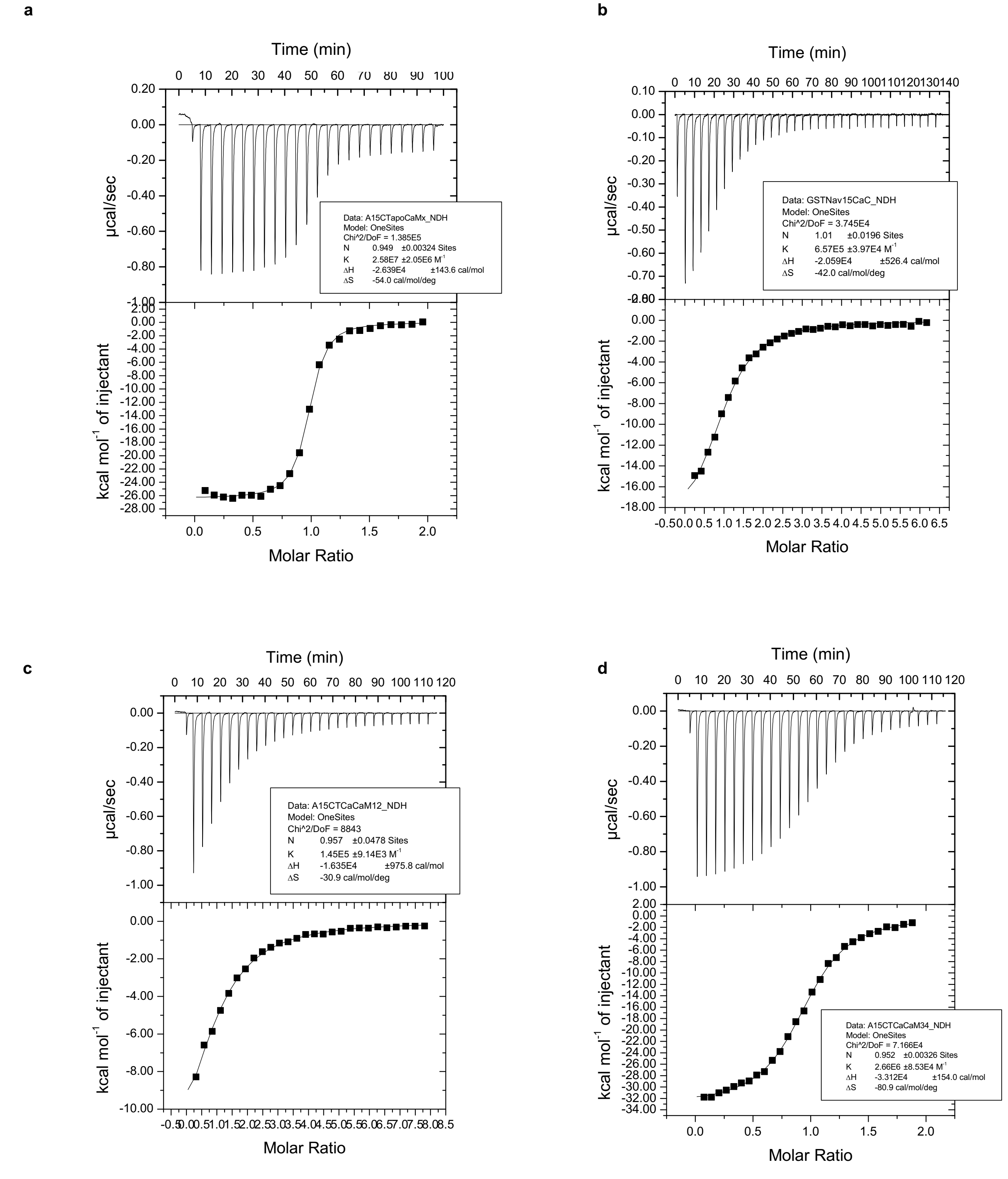

Supplementary Fig. 7 | Na$_V$1.5 CTerm Short and CaM Binding.
Isotherms of ITC titrations, top panel displays the heat evolved following each injection and the bottom panel shows the integrated heats of injection. **a,** apo CaM (64 µM) into Na$_V$1.5 CTerm short (6.8 µM). **b,** $(Ca^{2+})_4$-CaM (180 µM) into Na$_V$1.5 CTerm short (7 µM). **c,** $(Ca^{2+})_{2\text{-}C}$-CaM (336µM) into Na$_V$1.5 CTerm short (8 µM). **d,** $(Ca^{2+})_{2\text{-}N}$-CaM (81 µM) into Na$_V$1.5 CTerm short (8 µM).

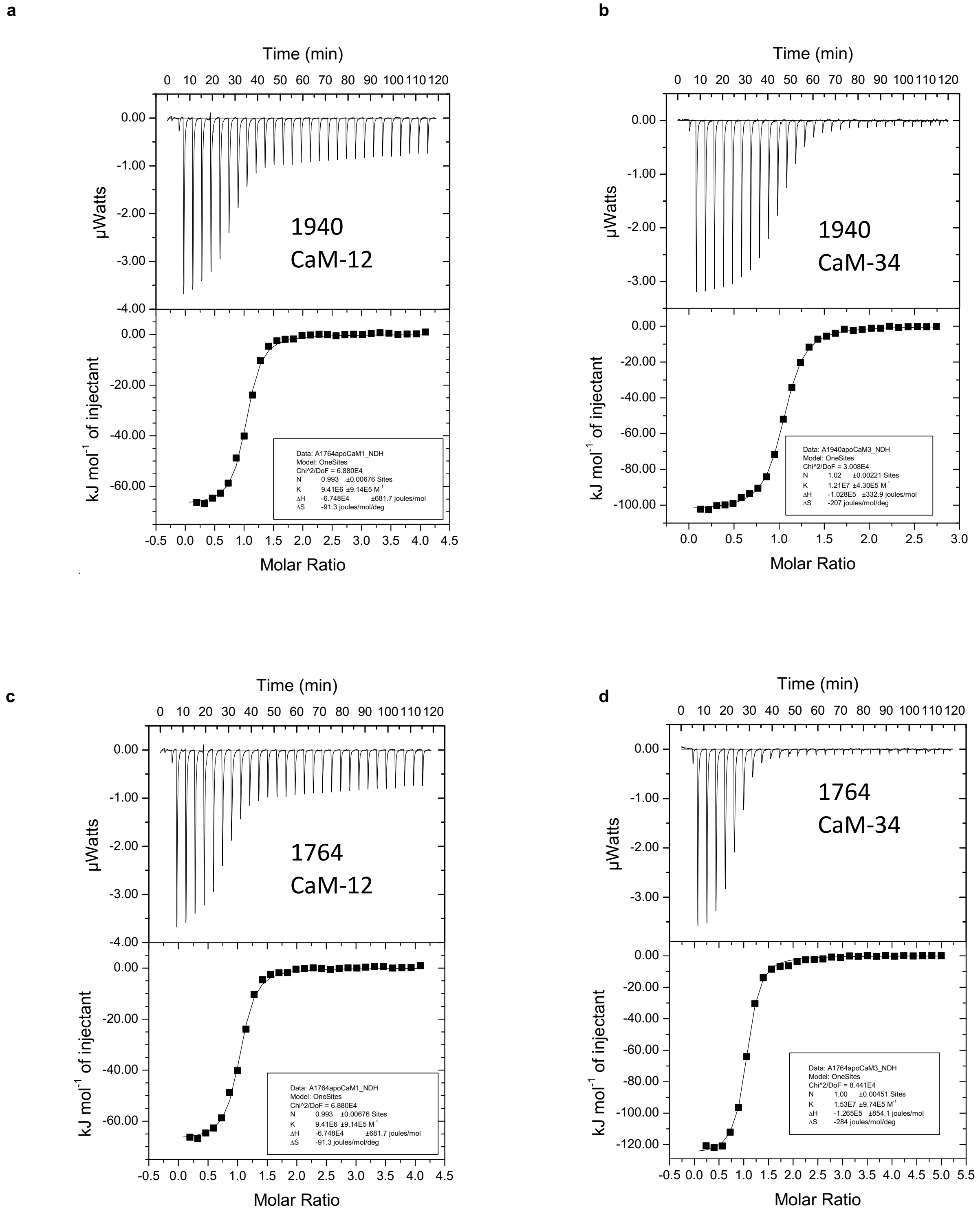

Supplementary Fig. 8 | apo CaM_{12} and apo CaM_{34} Binding to Na_V CTerm long. Isotherms of ITC titrations, top panel displays the heat evolved following each injection and the bottom panel shows the integrated heats of injection. **a,** apo CaM_{12} (81 µM) into Na_V1.5 CTerm long (8.5 µM). **b,** apo CaM_{34} (81 µM) into Na_V1.5 CTerm long (6.5 µM). **c,** apo CaM_{12} (81 µM) into Na_V1.4 CTerm long (6.3 µM). **d,** apo CaM_{34} (85 µM) into Na_V1.4 CTerm long (3.7 µM).

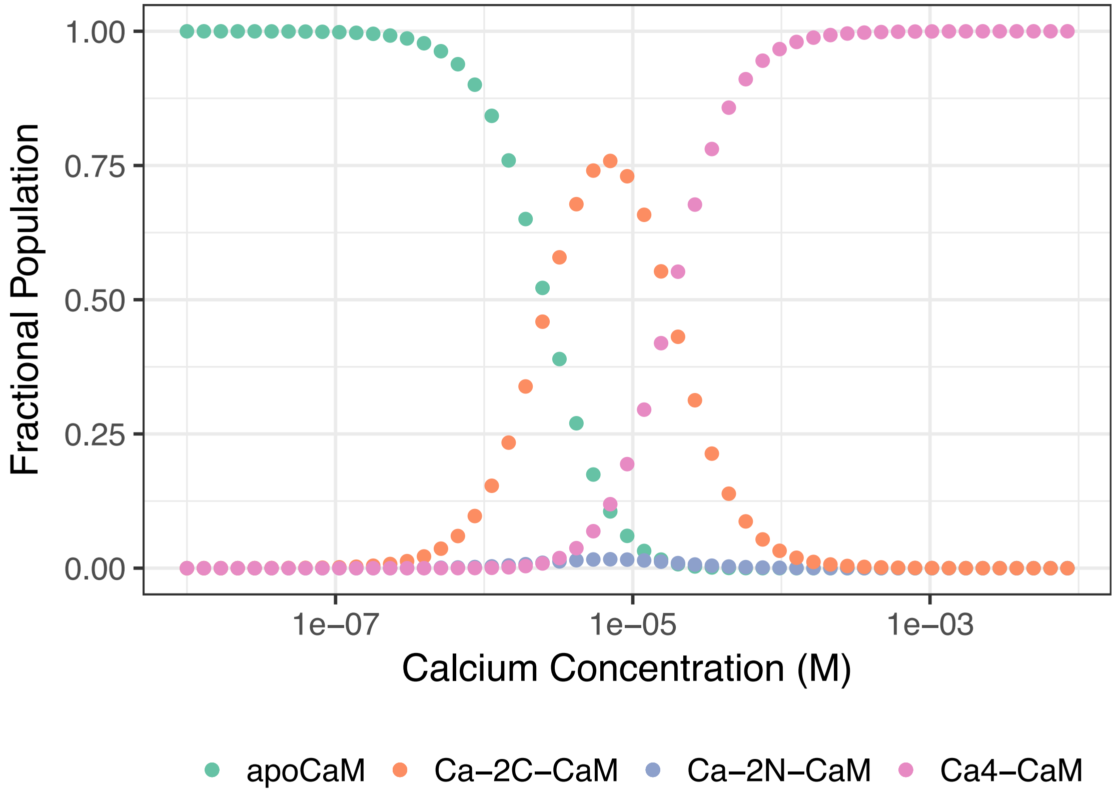

Supplementary Fig. 9 | CaM Population Model.
Fractional populations of free CaM species as a function of Ca^{2+} concentration. Calculated using the binding data reported by Evans *et al* used in the reaction scheme shown in Fig. 4a.[3]

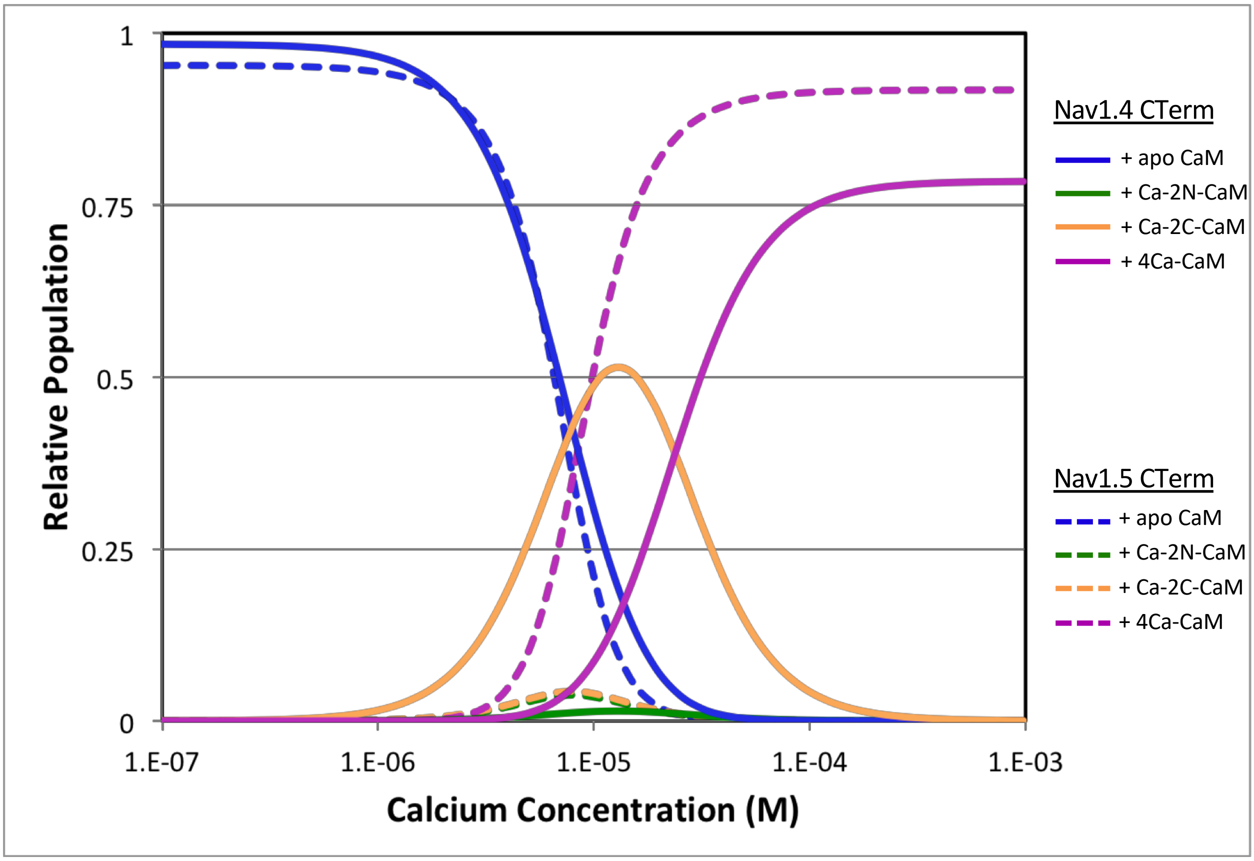

Supplementary Fig. 10 | Na_V CTerm-CaM populations as a function of Ca^{2+} at 1 μM CaM.

The relative populations of 4 CaM-bound CTerm species are shown at a fixed CaM concentration of 1 μM (each isoform CTerm species add to 100%, free CTerm is included in calculations but not shown in figure). The same trends as 10 μM CaM are seen: at ~10 μM Ca^{2+} $Na_V1.4$ CTerm shows a dominant species of $(Ca^{2+})_{2\text{-}C}$-CaM bound to CTerm (solid orange line) while $Na_V1.5$ CTerm shows a dominant species of $(Ca^{2+})_4$-CaM bound to CTerm (dashed purple line).

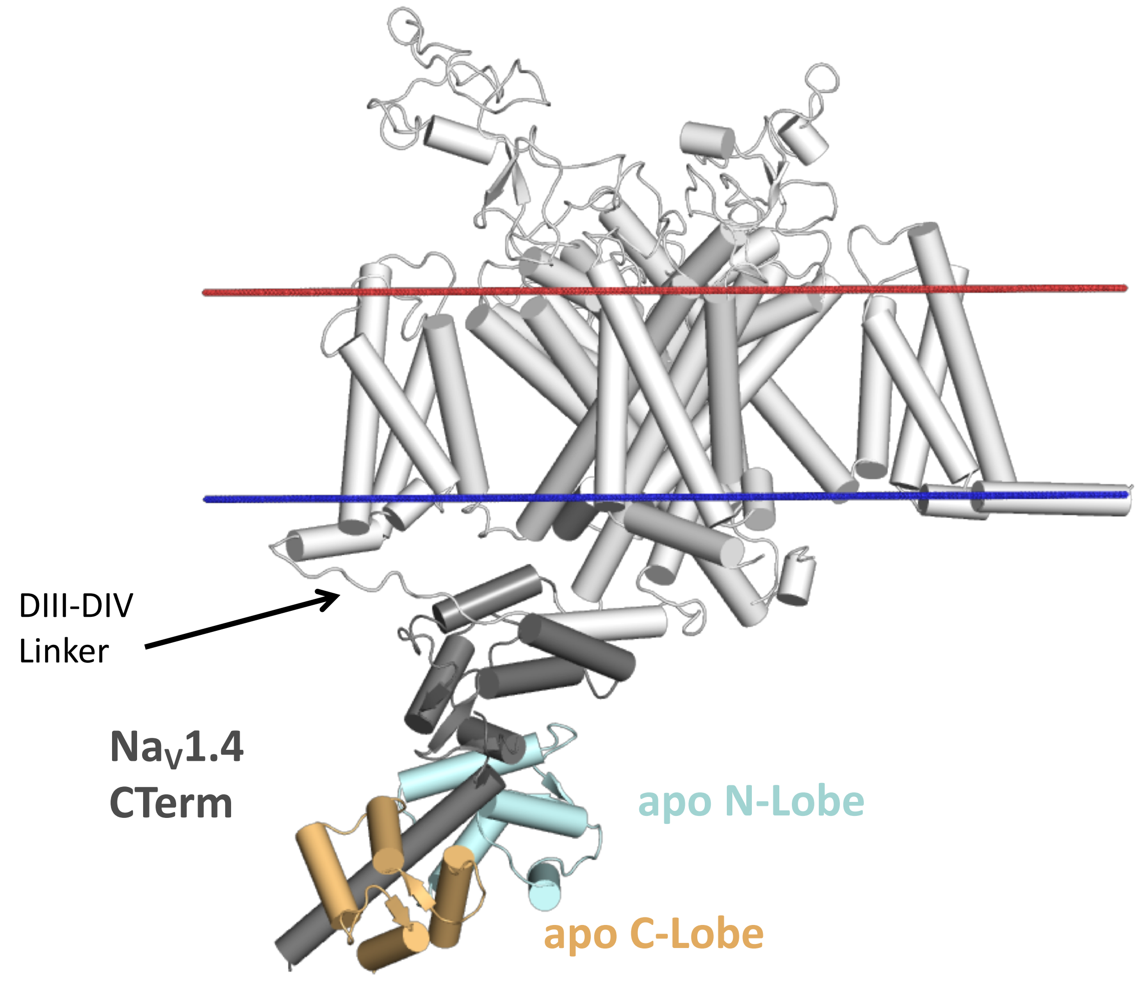

Supplementary Fig. 11 | Global topology of Nav1.4 with bound apo CaM. Homology model and crystal structure hybrid model; homology model in light gray built with Swiss Model[4] using NavPaS (PDB ID: 5XOM) [2] as template. Intracellular domains and loops not shown in model, with the exception of the DIII-DIV linker. Lipid membrane placed from OPM database[5] for 5XOM with red as extracellular and blue as intracellular edge. Crystal structure of $Na_V1.4$ CTerm and apoCaM overlaid by backbone alignment of CTerm helices 1-4 (residues 1615 to 1690, r.m.s.d. 0.66 Å, homology model residues not shown). CTerm is shown in dark gray and CaM lobes in teal (N-lobe) and light orange (C-lobe).

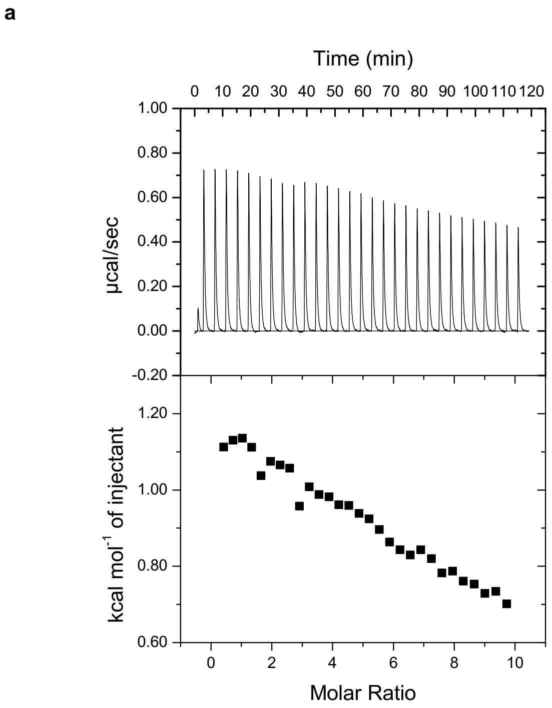

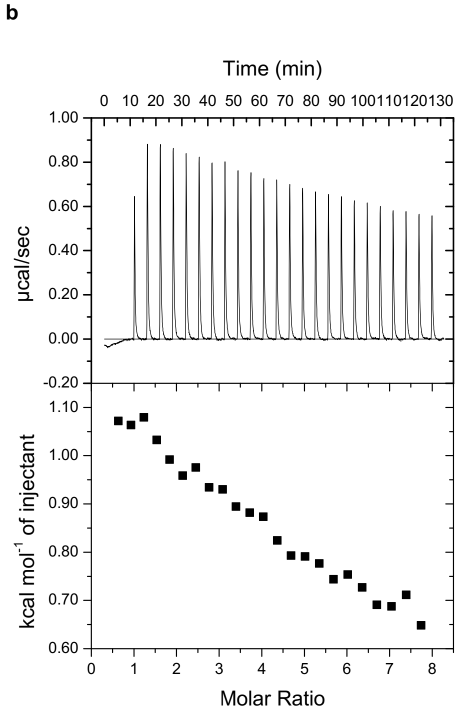

Supplementary Fig. 12 | DIII-DIV linker peptide titrations of Na$_V$1.4 CTerm and (Ca^{2+})$_4$-CaM complex. Isotherms of ITC titration, top panel displays the heat evolved following each injection and the bottom panel shows the integrated heats of injection. **a.** Titration of the DIII-DIV 12mer peptide (2 mM) into Na$_V$1.4 CTerm short and (Ca^{2+})$_4$-CaM (50 µM). Despite the high concentration of DIII-DIV linker use, no significant binding was observed. **b.** Titration of the DIII-DIV 20mer peptide (2 mM) into Na$_V$1.4 CTerm long and (Ca^{2+})$_4$-CaM (50 µM). Despite the high concentration of DIII-DIV linker use, no significant binding was observed.

a

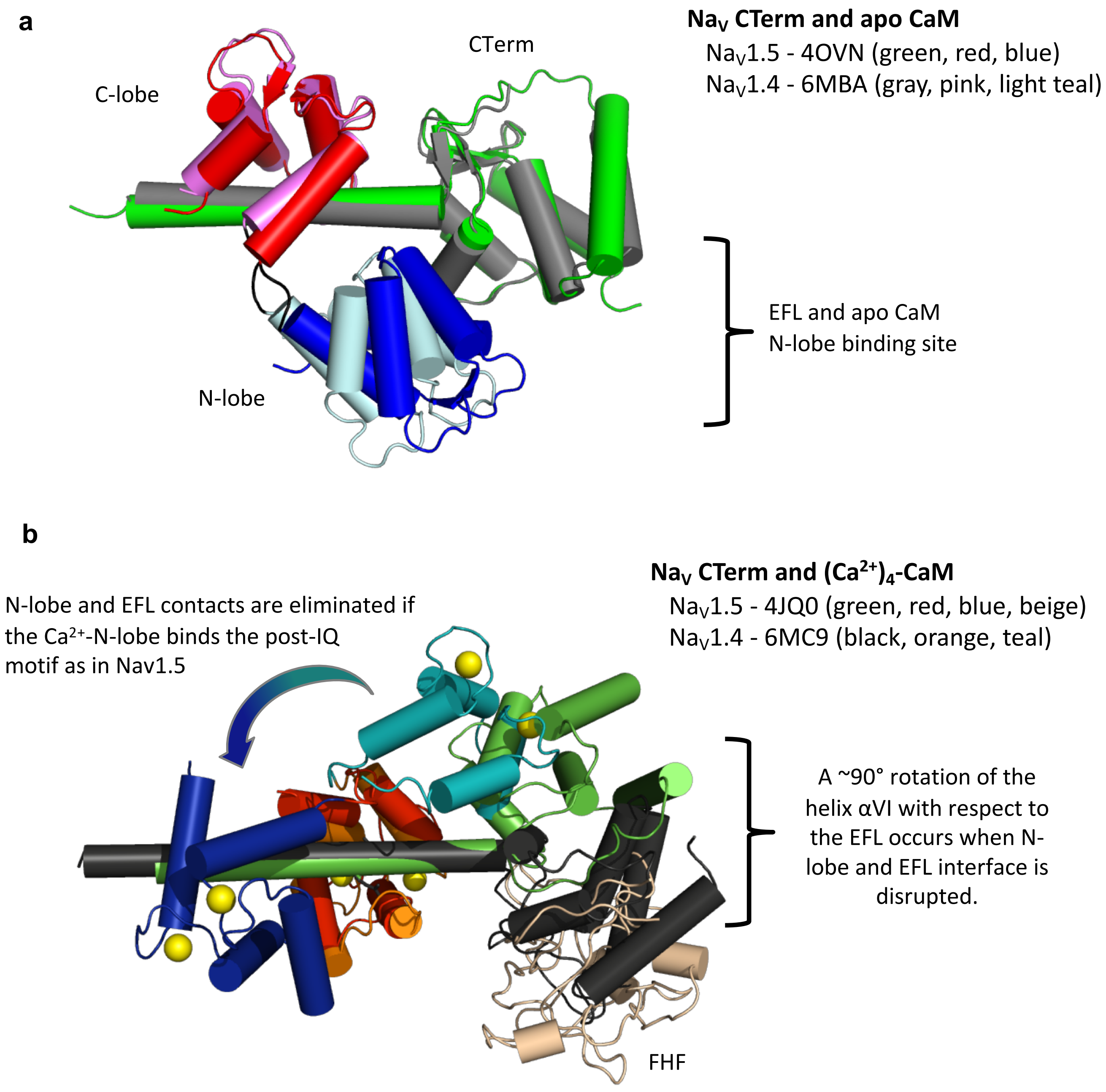

Supplementary Fig. 13 | Overlay of Nav1.4 and Nav1.5 CTerm and CaM complexes, ± Ca^{2+}. **a,** Overlay of Na$_V$1.5 CTerm and apo CaM (PDB ID: 4OVN chains D and I) with Na$_V$1.4 CTerm and apo CaM. Alignment by all atoms results in an r.m.s.d of 2.7 Å indicating the high similarity of the two structures. **b,** Overlay of Na$_V$1.5 CTerm, (Ca^{2+})$_4$-CaM and FHF (PDB ID: 4JQ0) with Na$_V$1.4 CTerm and (Ca^{2+})$_4$-CaM. The CaM-C-lobe is in the same relative position but the CaM N-lobe is seen contacting the EFL in Na$_V$1.4 and bound to the post-IQ motif in Na$_V$1.5. Additionally, a ~90° rotation of helix αVI relative to the EFL is seen in comparing the two structures. The FHF bound to Na$_V$1.5 CTerm is shown in beige.

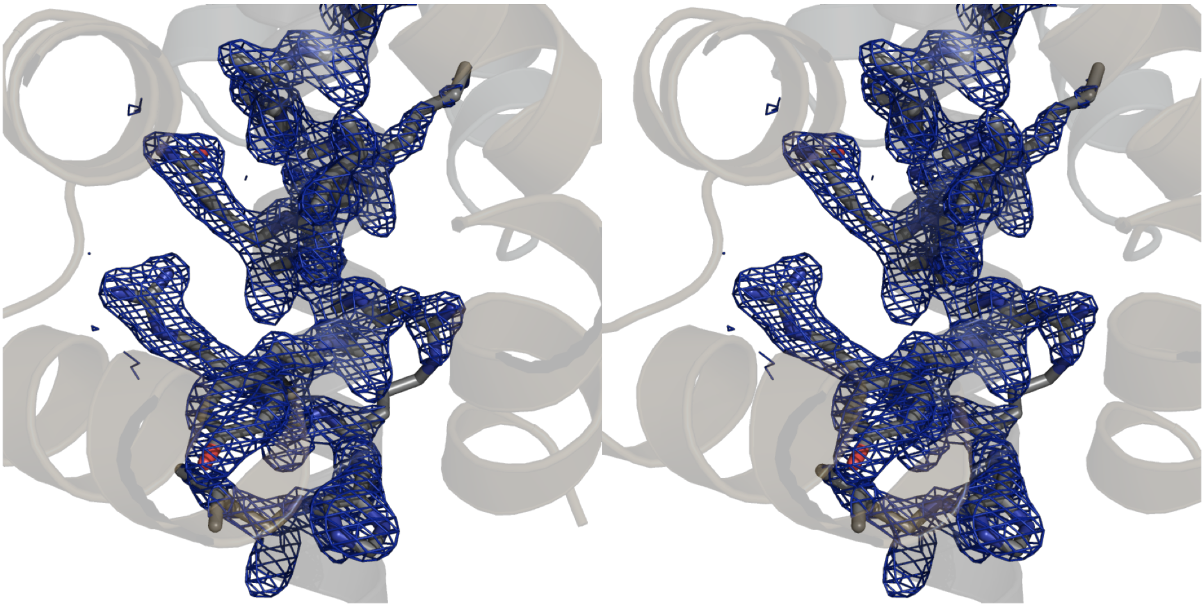

Supplementary Fig. 14 | Stereo image of electron density surrounding the IQ-motif of Na_V1.4 CTerm with apoCaM bound. The top-view of the IQ-motif and surrounding residues (Cys1730-Leu1742) of Na_V1.4 CTerm (gray) are shown as sticks with calmodulin C-lobe (light orange) shown as cartoon. The electron density map (blue mesh) is coutoured to 2.0 σ within 1.3 Å of the indicated Na_V1.4 CTerm residues.

Supplementary Table 1. Binding data of apo CaM_{12} and CaM_{34} species for $Na_V1.4$ or $Na_V1.5$ CTerm

Sample Cell	$[Ca^{2+}]$ mM	Titrant	Kd nM	-ΔG Kcal mol^{-1}
**Nav1.5 CTerm Long**	0	apo CaM_{12}	65±7	9.79
**Nav1.5 CTerm Long**	0	apo CaM_{34}	83±3	9.65
**Nav1.4 CTerm Long**	0	apo CaM_{12}	106±12	9.51
**Nav1.4 CTerm Long**	0	apo CaM_{34}	65±3	9.79

Supplementary Table 2. Electrophysiological measurements of CDI in Na_V1.5-CTail1.4 chimera

	(P2-P1)/P1 without Ca^{2+}	(P2-P1)/ P1 with Ca^{2+}
**Na_V1.5-CTail1.4 with WT CaM**	0.008815	-0.1788
	-0.03823	-0.18593
	-0.02721	-0.22115
	-0.02921	-0.3199
**Na_V1.5-CTail1.4 with CaM_{34}**	0.030079	-0.04126
	-0.01622	-0.01387
	0.033012	-0.10395
	-0.04639	-0.10615
	0.091316	-0.07427
	0.046462	-0.21015
	-0.01519	0.00021
**Na_V1.5-CTail1.4 with CaM_{12}**	0.035374	-0.01622
	-0.01695	-0.01531
	0.040256	0.044803

Supplementary Table 3. DNA oligonucleotide primers used for cloning.

	Sequence (5' - 3')
NaV1.5 ΔP-IQ cloning Forward (F)	AGAGCCAGTGTGAGTCCT
NaV1.5 ΔP-IQ cloning Reverse (R)	agctaatctagattaAGAGCGTTGCAGCAGG)
NaV1.4 1599-1754 Forward	ctgggatccgagaacttcaatgtg
NaV1.4 1599-1754 Reverse	tcgtctcgagttaggtacatgtaggatgcctgc

1. Kawasaki, H. & Kretsinger, R. H. Structural differences among subfamilies of EF-hand proteins--a view from the pseudo two-fold symmetry axis. *Proteins* **82**, 2915-2924, doi:10.1002/prot.24562 (2014).
2.Shen, H. *et al.* Structure of a eukaryotic voltage-gated sodium channel at near-atomic resolution. *Science* **355**, doi:10.1126/science.aal4326 (2017).
3.Evans, T. I. & Shea, M. A. Energetics of calmodulin domain interactions with the calmodulin binding domain of CaMKII. *Proteins* **76**, 47-61, doi:10.1002/prot.22317 (2009).
4.Waterhouse, A. *et al.* SWISS-MODEL: homology modelling of protein structures and complexes. *Nucleic Acids Res* **46**, W296-W303, doi:10.1093/nar/gky427 (2018).
5. Lomize, M. A., Pogozheva, I. D., Joo, H., Mosberg, H. I. & Lomize, A. L. OPM database and PPM web server: resources for positioning of proteins in membranes. *Nucleic Acids Res* **40**, D370-376, doi:10.1093/nar/gkr703 (2012).
