# Supplementary material for: Ca2+-dependent regulation of sodium channels NaV1.4 and NaV1.5 is controlled by the post-IQ motif
Source: Nat Commun. 2019 Apr 3;10:1514. doi: 10.1038/s41467-019-09570-7 (PMC6447637; doi:10.1038/s41467-019-09570-7)
Supplement: Supplementary file 2 — Reporting Summary [file 41467_2019_9570_MOESM2_ESM.pdf]

nature research

Corresponding author(s): L. Mario Amzel

Reporting Summary

Nature Research wishes to improve the reproducibility of the work that we publish. This form provides structure for consistency and transparency in reporting. For further information on Nature Research policies, see Authors & Referees and the Editorial Policy Checklist.

Statistical parameters

When statistical analyses are reported, confirm that the following items are present in the relevant location (e.g. figure legend, table legend, main text, or Methods section).

n/a	Confirmed	
☐	☒	The exact sample size (*n*) for each experimental group/condition, given as a discrete number and unit of measurement
☐	☒	An indication of whether measurements were taken from distinct samples or whether the same sample was measured repeatedly
☐	☒	The statistical test(s) used AND whether they are one- or two-sided<br>*Only common tests should be described solely by name; describe more complex techniques in the Methods section.*
☒	☐	A description of all covariates tested
☒	☐	A description of any assumptions or corrections, such as tests of normality and adjustment for multiple comparisons
☐	☒	A full description of the statistics including central tendency (e.g. means) or other basic estimates (e.g. regression coefficient) AND variation (e.g. standard deviation) or associated estimates of uncertainty (e.g. confidence intervals)
☐	☒	For null hypothesis testing, the test statistic (e.g. *F*, *t*, *r*) with confidence intervals, effect sizes, degrees of freedom and *P* value noted<br>*Give P values as exact values whenever suitable.*
☒	☐	For Bayesian analysis, information on the choice of priors and Markov chain Monte Carlo settings
☒	☐	For hierarchical and complex designs, identification of the appropriate level for tests and full reporting of outcomes
☒	☐	Estimates of effect sizes (e.g. Cohen's *d*, Pearson's *r*), indicating how they were calculated
☐	☒	Clearly defined error bars<br>*State explicitly what error bars represent (e.g. SD, SE, CI)*

Our web collection on statistics for biologists may be useful.

Software and code

Policy information about availability of computer code

Data collection	Crystallography data was collected at NSLS-II beamlines 17-ID-1 and 17-ID-2 using the beamline LSDC software.<br>ITC data was collected using VPViewer.<br>Electrophysiology data was collected with PCLAMP 10 and custom MATLAB scripts.
Data analysis	Crystallography data analysis and structure building was performed with XDS, ADXV, Fast DP, Pointless, Aimless, XScale, Coot, PyMol, Refmac, CCP4, Phenix, Phaser.<br>ITC data was analyzed with Origin 7. Thermodynamic modeling was performed with R.<br>Electrophysiology data was analyzed with Excel, Graphpad, Clampfit 10 and custom MATALAB scripts.

For manuscripts utilizing custom algorithms or software that are central to the research but not yet described in published literature, software must be made available to editors/reviewers upon request. We strongly encourage code deposition in a community repository (e.g. GitHub). See the Nature Research guidelines for submitting code & software for further information.

Data

Policy information about availability of data

All manuscripts must include a data availability statement. This statement should provide the following information, where applicable:

- Accession codes, unique identifiers, or web links for publicly available datasets
- A list of figures that have associated raw data
- A description of any restrictions on data availability

6MBA, 6MC9

Field-specific reporting

Please select the best fit for your research. If you are not sure, read the appropriate sections before making your selection.

☒ Life sciences ☐ Behavioural & social sciences ☐ Ecological, evolutionary & environmental sciences

For a reference copy of the document with all sections, see nature.com/authors/policies/ReportingSummary-flat.pdf

Life sciences study design

All studies must disclose on these points even when the disclosure is negative.

Sample size	For ITC data multiple titrations (2-4) were run for each measurement and a representative isotherm is reported.<br>For electrophysiology n represents the total number of cells from which the data were obtained.
Data exclusions	No data were excluded from analysis.
Replication	See above.
Randomization	N/A
Blinding	N/A

Reporting for specific materials, systems and methods

Materials & experimental systems

n/a	Involved in the study
☐	☒ Unique biological materials
☒	☐ Antibodies
☐	☒ Eukaryotic cell lines
☒	☐ Palaeontology
☒	☐ Animals and other organisms
☒	☐ Human research participants

Methods

n/a	Involved in the study
☒	☐ ChIP-seq
☒	☐ Flow cytometry
☒	☐ MRI-based neuroimaging

Unique biological materials

Policy information about availability of materials

Obtaining unique materials	Constructs for all unique materials used are available from the authors upon request.

Eukaryotic cell lines

Policy information about cell lines

Cell line source(s)	American Type Culture Collection (ATCC)
Authentication	Obtained from vendor, no further authentication performed.

Mycoplasma contamination	All Eukaryotic cell lines tested negative for Mycoplasma contamination.
Commonly misidentified lines<br>(See ICLAC register)	No commonly misidentified cell lines were used.
